# Mechanisms of simultaneous linear and nonlinear computations at the mammalian cone photoreceptor synapse

**DOI:** 10.1038/s41467-023-38943-2

**Published:** 2023-06-16

**Authors:** Chad P. Grabner, Daiki Futagi, Jun Shi, Vytas Bindokas, Katsunori Kitano, Eric A. Schwartz, Steven H. DeVries

**Affiliations:** 1 Institute for Auditory Neuroscience, University Medical Center Göttingen, 37075 Göttingen, Germany; 2 Synaptic Nanophysiology Group, Max Planck Institute for Multidisciplinary Sciences, 37077 Göttingen, Germany; 3 College of Information Science and Engineering, Ritsumeikan University, Shiga, Japan; 4 Center for Systems Visual Science, Organization of Science and Technology, Ritsumeikan University, Shiga, Japan; 5 Ritsumeikan Global Innovation Research Organisation, Ritsumeikan University, Shiga, Japan; 6 Department of Ophthalmology, Northwestern University Feinberg School of Medicine, Chicago, IL 60611 USA; 7 Dept of Pharmacological and Physiological Sciences, The University of Chicago, Chicago, IL 60637 USA

**Keywords:** Retina, Neurotransmitters

## Abstract

Neurons enhance their computational power by combining linear and nonlinear transformations in extended dendritic trees. Rich, spatially distributed processing is rarely associated with individual synapses, but the cone photoreceptor synapse may be an exception. Graded voltages temporally modulate vesicle fusion at a cone’s ~20 ribbon active zones. Transmitter then flows into a common, glia-free volume where bipolar cell dendrites are organized by type in successive tiers. Using super-resolution microscopy and tracking vesicle fusion and postsynaptic responses at the quantal level in the thirteen-lined ground squirrel, *Ictidomys tridecemlineatus*, we show that certain bipolar cell types respond to individual fusion events in the vesicle stream while other types respond to degrees of locally coincident events, creating a gradient across tiers that are increasingly nonlinear. Nonlinearities emerge from a combination of factors specific to each bipolar cell type including diffusion distance, contact number, receptor affinity, and proximity to glutamate transporters. Complex computations related to feature detection begin within the first visual synapse.

## Abstract (web summary)

Cone photoreceptors signal to several bipolar cell types at one of the most structurally complex synapses in the central nervous system. Here, the authors show how the 3D organization of this synapse creates different signals in the postsynaptic OFF bipolar cell types.

## Introduction

At the compact cone synapse, graded changes in membrane voltage are encoded by ~20 ribbon active zones^1–3^ as a spatiotemporal pattern of vesicle fusion. The resulting glutamate concentration profile in the synaptic cleft is then sampled and re-encoded as electrical signals by the dendritic contacts of more than a dozen types of On and Off bipolar cells (BCs)^4–10^. In daylight, when photon flux is high, cone membrane voltage is a continuous representation of illumination with increases causing membrane hyperpolarization and decreases causing depolarization. At individual cone ribbons, a close association between L-type Ca^2+^ channels^11,12^ and membrane docked vesicles enables a probabilistic stream of fusion events whose rate is governed by membrane voltage^13,14^ and the kinetics of vesicle replenishment^15,16^. Under conditions where light intensity fluctuates modestly about a mean value and rates of replenishment and release are in balance, the Ca^2+^ nanodomains that link individual channel opening to local vesicle fusion create a linear relationship between membrane voltage and transmitter release^13,14,17^. More extreme, but still physiological, changes in illumination can produce a synchronous release whose amplitude depends on both the present stimulus and stimulus history, a nonlinearity that results from an imbalance between ultrafast docked vesicle pool depletion and slow replenishment^15,18,19^. Unlike in inner hair cells where each ribbon maintains an exclusive relationship with a postsynaptic contact^20,21^, the transmitter released at cone ribbons flows first over nearby BC dendrites and then into a common glia-free space containing hundreds of BC dendrites^1,22,23^. The potential for both exclusive and non-exclusive relationships between ribbons and postsynaptic sites raises the possibility that different contact sites could be specialized to transduce different components of cone transmitter output. Here, under conditions that simulate both low amplitude continuous and larger amplitude transient release, we test whether there is a 1:1 relationship between transmitter release and dendritic response, which is typical of active zones and post-synaptic densities at many central excitatory synapses^24,25^ and compatible with low amplitude linear transmission at the cone synapse, or whether a postsynaptic response requires overlapping release from multiple ribbons, a fundamentally nonlinear mechanism that may favor responses to larger, transient components in the cone output^26–28^.

**Fig. 1:**
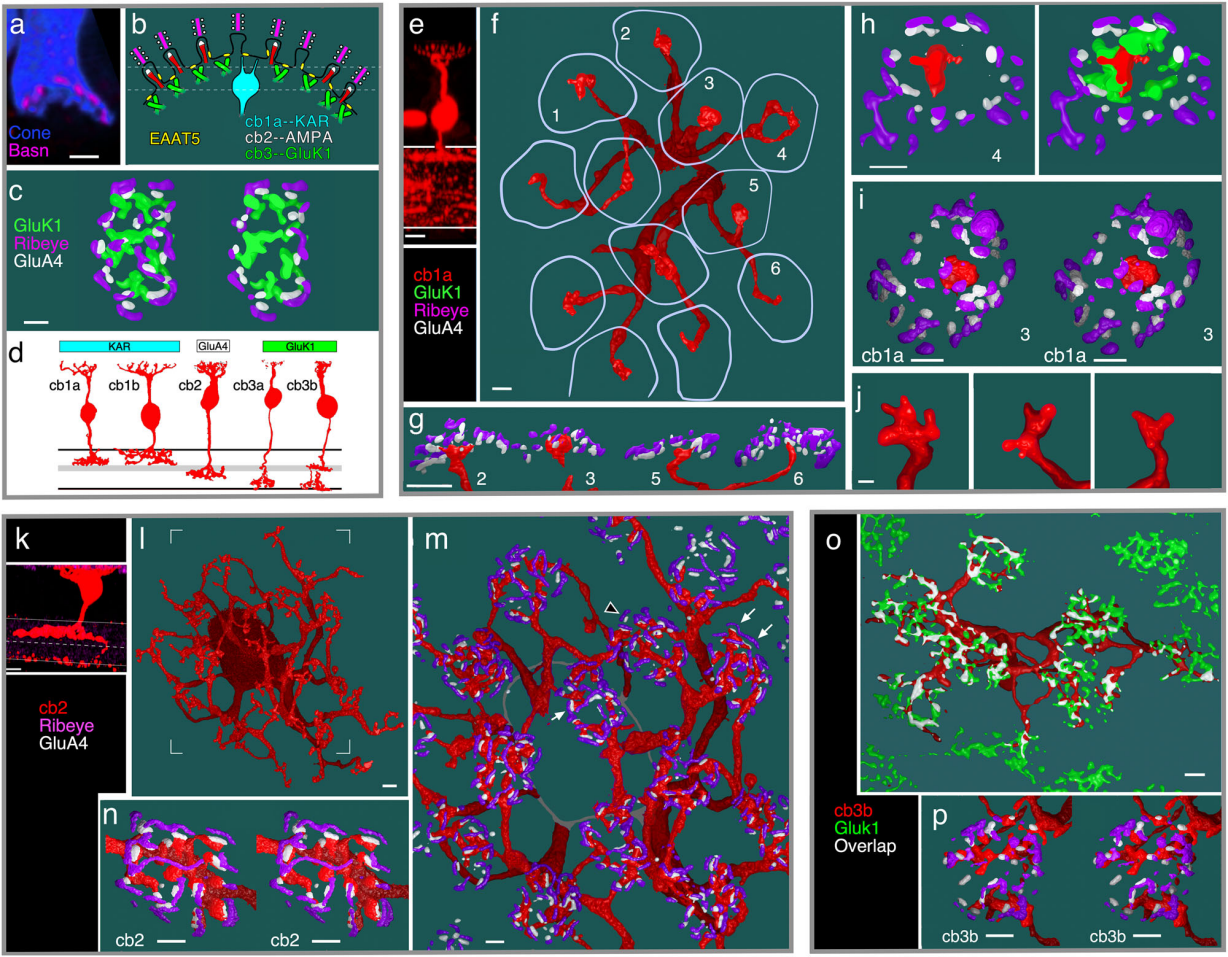
Cone contacts of the different off BC types. **a** Vertical section through a cone terminal filled with Cascade blue and immunolabeled with an antibody to bassoon (Basn) showing a concave shape (similar results from 5 cones in 2 retinas). **b** Summary illustration of labeled structures at the cone synapse including ribbons (magenta) atop membrane invaginations, invaginating cb2 cell dendrites (red) with tip-associated GluA4-labeling (white), GluK1 labeling beneath the terminal (green), the central location of kainate receptor (KAR) expressing cb1a cell contacts (cyan), and clusters of EAAT5 transporters in the cone terminal membrane (yellow). **c** En face view of a labeled cone terminal (left) and cross-sectional slab (right) at the level of the dashed lines in (**b**) showing the concentric nested organization of Ribeye, GluA4, and GluK1 (similar results from multiple cones in 5 retinas). **d** Morphology of ground squirrel Off BC types and their predominant glutamate receptor subunits. Adapted from Light, A. C. et al. Organizational motifs for ground squirrel cone bipolar cells. J Comp Neurol 520, 2864-2887 (2012). **e–j** Cb1a cell cone contacts. **e** Confocal image of a GFP-labeled cb1a cell. **f**. 3D STED microscopic reconstruction of the cb1a cell in the whole mount configuration with boundaries of overlying cone terminals (lavender; similar results from 4 cb1a cells in 3 retinas). **g** Diagonal cross-section showing centrally located cb1a cell contacts relative to Ribeye and GluA4 clusters. **h** Tangential section of terminal 4 without (left) and with (right) GluK1 labeling. **i** Stereo image of terminal 3. **j** Select dendritic endings from a different cb1a cell (scale = 0.5 µm; 2 similar cells from 2 retinas). **k–n** Cb2 cell cone contacts. **k** Confocal image of a GFP-labeled cb2 cell. **l** En face view of the cb2 cell. **m** Zoomed-in view of the cb2 cell showing the close juxtaposition between Ribeye and GluA4 (arrowhead) and cb2 cell dendrites (arrows; similar results from 6 cb2 cells in 4 retinas). **n** Stereo image of a cone terminal from a different cb2 cell. **o**, **p** Cb3 cell cone contacts. **o** GFP-labeled cb3 cell showing overlap with gluK1 labeling (similar results from 5 cb3a/b cells in 3 retinas). **p** Stereo image of one cone terminal. Scale bar for confocal images = 5 µm. Scale bar = 1 µm unless specified.

The ultrastructure of the cone synapse provides a substrate for diverse signaling mechanisms. Cones make two specialized synaptic contacts: invaginations and basal junctions^29,30^. Approximately twenty membrane invaginations, each 200–400 nm deep and marked at their apex with a ribbon^23^, open onto a ~3 µm diameter cone terminal base (Fig. 1a, b). In mammals, with few exceptions^31,32^, individual dendrites from On BCs enter cone invaginations and end in a 1:1 relationship with a ribbon active zone^33,34^. Off cb2 BCs in the ground squirrel are also functionally invaginating^35^. In contrast, the vast majority of Off BC types contact the base of the cone terminal^36^, which lacks the specializations that are typically associated with transmitter release^37–39^. Basally contacting Off bipolar cells can be subdivided into types that tend to contact near the mouth of invaginations or between invaginations^34,36^. An obligatory diffusion distance of 250-700 nm from ribbon sites to basal junctions challenges the classical notion that individual quanta are the basis for synaptic transmission. Additionally, the location of basal dendritic contacts between invaginations raises the possibility that a contact can be excited by near simultaneous release from nearby invaginations.

By using 3D STED microscopy to reconstruct synaptic contacts and by directly counting the number of vesicles released by a cone while simultaneously measuring the responses in postsynaptic BCs, we show that the individual quantum is not the basis for neurotransmission for most Off BC types. Rather, different Off BC types respond to different spatiotemporal groupings of events, conferring sensitivity to patterns in the synaptic vesicle stream.

## Results

3D STED microscopic reconstructions show the nested, concave shape of the thirteen-lined ground squirrel, *Ictidomys tridecemlineatus*, cone terminal. Synaptic ribbons in the outermost shell were localized with antibodies against either the active zone protein bassoon^40^ or the ribbon protein Ribeye (Fig. 1a–c)^41^. GluA4, expressed by ground squirrel cb2 BCs and to a lesser extent horizontal cells^42,43^, labeled central to Ribeye/bassoon. Innermost was GluK1, which is expressed by cb3a/b BCs^42^. Ground squirrel retina also contains cb1a and cb1b BCs^6^ (Fig. 1d) whose kainate receptor (KAR) subunit composition is unclear^42^. We reconstructed the dendritic trees of representative cb1a, cb2, and cb3b cells that were individually labeled with GFP through viral transduction^6^ (Fig. 1e–p). Cb1a cell dendrites each had a prominent bulb-like expansion that approached the central region of the cap-shaped cone terminal base (Fig. 1f–i) and ended in 2-5 short, fine processes (Fig. 1j; Supplementary Fig. 4k–l). In contrast, cb2 cell dendrites made multiple contacts that frequently ended near clusters of GluA4 subunits which, in turn, were juxtaposed to Ribeye (Fig. 1k–n), consistent with an invaginating locus. The dendrites of cb3 cells (Fig. 1o, p) made multiple cone contacts that colocalized with GluK1 labeling, consistent with a basal localization. The different contact patterns of cb1a, cb2, and cb3 cells prompted us to determine whether the Off BC types sampled the population of ribbon release sites in different ways.

### Measuring ribbon-mediated vesicle fusion at cone terminals

**Fig. 2:**
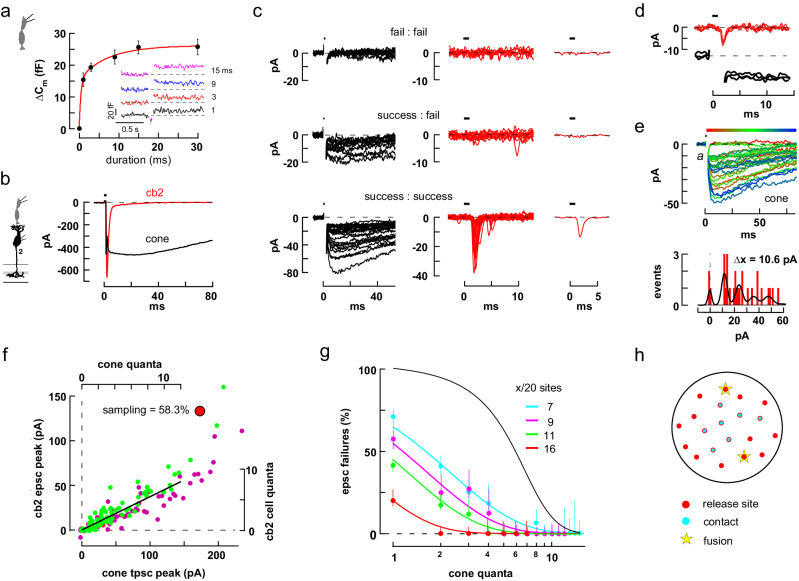
Cone to cb2 cell synapses are linear. **a** Plot of ΔC_m_ versus step (–70 to –10 mV) duration fitted with a bi-exponential curve (*n* = 7 independent cones; mean ± S.E.). *Inset*. Individual responses. **b** Maximal pre- and postsynaptic currents elicited by a brief cone depolarization (horizontal bar). **c** Grouped traces selected from a train of 98 consecutive cone depolarizations to either -35 or -30 mV. Upper row. Left. Superimposed cone response failures. Middle. Corresponding BC traces. Right. Averaged BC response consistent with an absence of events. Middle row. Pairs selected for cone response accompanied by BC failure. Bottom row. Larger cone tpscs with corresponding BC epscs. Upper and middle rows show all responses that met the criteria. For clarity, the lower row shows one-quarter of the responses. **d** Three trace pairs showing small, near-identical responses in the cone and BC. **e** Consecutive tpsc responses following 1 ms steps from -70 to -30 mV color-coded according to temporal sequence (above). Amplitude histogram with sum-of-Gaussians fit (below). **f** Plot of BC peak epsc versus cone peak tpsc amplitude. Green (steps to –35) and purple (to –30 mV) circles encompass the traces in *c*; red circles plot responses during steps to –40 mV. Axes (top and right) were scaled by unitary event amplitudes in the cone and BC. A straight-line fit to the scaled data had a y-intercept of 0.039 quantal units. Large red circle denotes the corresponding data points in Fig. 2g. **g** Epsc response failures plotted against tpsc quantal content for 4 independent cone to cb2 cell pairs and fitted with theoretical curves (see “Methods”). The black curve shows the limiting case in which all release sites are sampled and a success requires ≥2 released vesicles at any site. Confidence intervals (50%) are related to trial number and failure probability (see “Methods”). **h** Cone terminal model for cb2 responses showing 20 release sites, x = 7 of which are contacted by dendrites. Vesicles randomly fusing at 2 sites (e.g., stars) produce response failures with a probability of 0.42. Source data for this and subsequent figures are provided as a Source Data file. Silhouettes republished from Neuron, Vol 91, Grabner, C. P. et al. Mechanism of High-Frequency Signaling at a Depressing Ribbon Synapse, 133–145, (2016), with permission from Elsevier.

We first wanted to verify that a brief, 1 ms cone depolarization elicits exocytosis from a ribbon-associated and membrane-docked vesicle pool, rather than some other membrane-associated pool. We used the relationship between the change in membrane capacitance (ΔC_m_) and step duration to determine the number of vesicles in the readily releasable pool (RRP), and then compared this number to the number of ribbon- and membrane-associated vesicles as counted by EM. Capacitance measurements showed that a 1 ms cone membrane voltage step caused a change of ~16 fF while longer steps, up to 30 ms, led to incremental gains (Fig. 2a). A plot of average ΔC_m_ versus step duration was best fit with a bi-exponential curve with τ_f_ = 0.4 ± 0.2 and τ_s_ = 7.3 ± 3.8 ms with amplitudes of 15.6 ± 3.0 and 10.5 ± 2.5 fF, respectively (mean±S.E.; r = 0.98; *n* = 7 cones). Assuming a mean single vesicle capacitance of 43 attofarads (Supplementary Fig. 1k), ultrafast and fast release pools^44^ contained ~360 and ~240 synaptic vesicles (SVs) creating an RRP of ~600 SVs. For comparison, based on electron microscopic reconstructions (Supplementary Fig. 1), we calculated that a two-sided rectangular ribbon held 114 SVs with 29 positioned at the base. STED microscopic images showed 22.1 ± 2.7 active zones per cone terminal (*n* = 10 terminals from 2 retinas) giving a total of ~640 vesicles associated with the cone membrane at active zones. The close correspondence between the physiologically measured RRP, 600 SVs, and the ultrastructurally measured ribbon-docked pool, 640 SVs, suggests that short depolarizing pulses elicit release from ribbon-docked pools^45^.

Insofar as whole-cell capacitance measurements cannot readily detect single fusion events, we used the cone glutamate transporter presynaptic current (tpsc) to measure transmitter release during brief depolarizations that caused only a few vesicles to fuse. Released glutamate feeds back onto the presynaptic cone terminal to elicit a large-amplitude, long-lasting inward current that is mediated by an anion conductance intrinsic to a TBOA-sensitive glutamate transporter (Fig. 2b)^46,47^. In a typical experiment, a train of 1 ms pulses (Fig. 2e) elicited cone tpscs that were interspersed with failures. Estimates of unitary event amplitude were obtained both by fitting an amplitude histogram with a sum of Gaussians (Fig. 2e; Supplementary Fig. 2a) and by calculating the probability of response failure, P_0_, during steps to relatively hyperpolarized voltages (Supplementary Fig. 2b, c). Cone tpscs provided a sensitive measurement of transmitter release at low levels, but began to saturate at amplitudes that exceeded ~35% of their maximal value (Supplementary Fig. 2d) or 150–200 pA (~12 quanta). Transporter saturation at moderate levels of glutamate release was supported by rapid perfusion experiments where steps into 20 µM glutamate produced near maximal transporter currents in isolated and voltage-clamped cones (Supplementary Fig. 2e, f). In our experiments, quantitation of the number of released quanta at the cone terminal was limited to the sub-saturating range.

### Cb2 cells detect single quantal events and respond linearly

We compared the amount of transmitter released by a presynaptic cone to the corresponding response in a postsynaptic cb2 BC. On the idea that GluA4 receptors on an invaginating cb2 cell dendrite respond exclusively to individual fusion events at a juxtaposed ribbon, and that the responses in the individual dendrites sum at the nearby soma, we hypothesized a linear relationship between tpsc and epsc amplitudes at low levels of release with a slope related to the fraction of cone ribbon active zones sampled. Stepping a cone from –70 to –20 mV for 1 ms produced maximal peak responses in a cone and cb2 cell (Fig. 2b). We then applied a train of weak depolarizing pulses to the cone and grouped the current responses in the cone and BC into three sets for illustration. First, response failures in the cone were always associated with failures in the cb2 BC (Fig. 2c, top row). The opposite behavior, namely, BC events during cone response failures, would suggest incomplete reporting by the cone terminal. Second, we selected for the simultaneous occurrence of a cone response and a BC failure (Fig. 2c, middle row). Cone responses and BC failures can occur if some cone release events are not detected by the postsynaptic cb2 cell. Evidence for postsynaptic sensitivity to individual fusion events comes from trace pairs in the series in which the simultaneous pre- and postsynaptic responses consisted of small events with stereotyped amplitudes (Fig. 2d). Finally, larger cone presynaptic responses were invariably associated with postsynaptic responses (Fig. 2c, lower row). The occasional failure to detect release is consistent with the idea that a cb2 cell does not contact every cone invagination and responds only to local quantal release, at least at low stimulus levels.

To determine the fraction of presynaptic sites sampled by a cb2 cell, we plotted the peak amplitude of each BC epsc against the peak of its corresponding cone tpsc (Fig. 2f). The resulting scatterplot was fitted by a straight line for responses in the cone-linear range (i.e, 12 quanta or about –130 pA for this pair). Cone responses beyond this range were associated with a dramatic increase in the BC response amplitude up to the maximum shown in Fig. 2b, a consequence of transporter saturation (Supplementary Fig. 2d–f). When responses were normalized by the unitary amplitudes (Fig. 2e for the cone and 6.75 pA for the cb2^35^), a straight-line fit (Fig. 2f) passed close to the origin, consistent with single event sensitivity in the BC. The normalized linear fit had a slope of 0.583 suggesting that ~60% of the cone’s ribbon release sites were sampled by the cb2 cell’s dendrites. Over the linear region of the plot, a fit to a power-law function, y=*a*x^b^ + c, had an exponent, b, of 0.94 which is close to one (the linear model is preferred, *p* = 0.7079; F test versus comparison equation y=*a*x + c; similar results were obtained in 7 out of 10 cone to cb2 cell pairs; Supplementary Fig. 3a–e). The average sampling of cone release sites measured using the scatterplot method was 49.3 ± 6.8% (±S.E., *n* = 10 pairs) with a y-intercept of -0.25 ± 0.07 quanta (*n* = 9, mean±S.E.; excluding one statistical outlier of –4.72 quanta).

We used a second approach to determine the fraction of cone release sites sampled by a cb2 cell. In this approach, we assigned an effective quantal content to each tpsc irrespective of the cone voltage step during an experiment and then plotted the percentage of BC response failures as a function of quantal content (Fig. 2g). The second approach made no assumptions about BC quantal response size. Plots of percent response failure verses the number of cone vesicles released were fitted with ideal curves obtained by assuming (1) a fixed number of invaginating sampling sites relative to 20 ribbon active zones; (2) that invaginating dendrites respond to local release at the single quantal level; and (3) that the probability of release at different ribbons is similar (Fig. 2h). Using this approach, the cb2 cell in Fig. 2b–f sampled 80% of the cone’s release sites (Fig. 2g, red circles). In 8 of the experiments, contact numbers ranged from 4 to 16 out of 20 (40.6 ± 6.6% sampling; mean ± S.E.; see also Supplementary Fig. 3f). More generally, a mid-range slope of 1 which resulted when the data was plotted semi-logarithmically suggests that detection is fundamentally binary, with the fusion of one or more glutamate containing vesicles at a contact associated with success and the fusion of no vesicles at a contact with failure. The two analytical approaches produced comparable results (Supplementary Fig. 3g).

As a check on the physiological measurements of the fraction of release sites sampled, we used STED microscopic reconstructions to count the fraction of ribbon sites contacted by cb2 cell dendrites in overlying cone terminals (Fig. 1m and Supplementary Fig. 3h). Focusing on central cones in a dendritic field, which are typically innervated by a single cb2 cell^9^, we obtained a contact fraction of 80.7 ± 4.7% (*n* = 2 cb2 cells with 19.0 ± 4.4 presynaptic cones per cell), comparable to the maximal value of 60–80% found from paired recordings.

### Sampling by cb1 and cb3 BCs

**Fig. 3:**
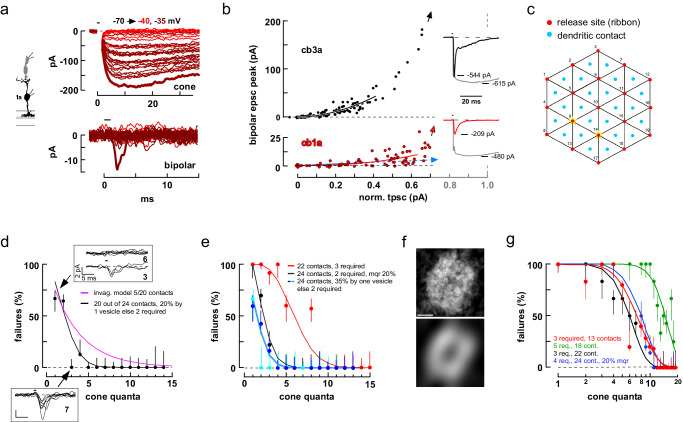
Cone to cb1 and cb3 cell synapses are nonlinear. **a** Consecutive responses in a cone to cb1a cell pair during two stimulus epochs. **b** Scatterplot of cb3a (upper panel) and cb1a (lower panel) epsc versus normalized cone tpsc amplitude. Red-brown circles include the responses in (**a**). Data points were fitted with a power-law function over the cone-linear range (cb1a: exponent equaled 3.26 ± 0.48, *n* = 8; cb1b: 2.00 ± 0.12, *n* = 2, not significantly different, *p* = 0.2436, mean ± S.E.; cb3a: 2.16 ± 0.17, *n* = 10; cb3b, 2.33 ± 0.12, *n* = 11, not significantly different, *p* = 0.4374; two-tailed unpaired *t* test). *Insets*. Maximal cone tpscs and BC epscs. Arrows point to the location of the maximal BC epsc response on the 100% cone response perpendicular (gray dashed line). **c** Synapse model incorporating 19 release sites and up to 24 basal contacts. **d** Plot of BC response failure as a function of cone tpsc quantal content for the cb3a cell in (**b**). *Insets*. Individual responses when quantal content equaled one (6 failures and 3 successes) and three (0 and 7). The best matching theoretical curve from the model in Fig. 2g, h (magenta; 5 contacts out of 20 sites). The black curve is from the model in (**c**) where a success occurs when an individual contact is exposed to two nearby simultaneous release events and the BC samples release from 20 out of 24 cone contact sites. The model is further adjusted so that 20% of the uniquantal release events produce a success. **e**. Failure plots for 4 independent cone to cb3 cell pairs. The steep decay in one of the plots (black circles) was better fitted by assuming that 20% of the release events were multiquantal (mqr). In one pair, the fit cooperativity was 3. **f** GluK1 labeling in a collapsed 3D image stack was binarized and individual terminals were digitally excised, point-by-point averaged (upper panel) and smoothed (lower panel; original data from Fig. 1o; see Supplementary Fig. 6). Scale = 1 µm. **g** Plots of response failure versus cone tpsc quantal content for 4 independent cone to cb1a cell pairs. Red points correspond to the data shown in (**a**, **b**). Confidence intervals are 50%. BC silhouette republished from Light, A. C. et al. Organizational motifs for ground squirrel cone bipolar cells. J Comp Neurol 520, 2864-2887 (2012). Cone silhouette republished from Neuron, Vol 91, Grabner, C. P. et al. Mechanism of High-Frequency Signaling at a Depressing Ribbon Synapse, 133-145, (2016), with permission from Elsevier.

We performed similar experiments on cone to cb1 cell pairs. Unlike in cone to cb2 cells pairs, where the BC response increased in proportion to the number of released vesicles, the cb1 cell response displayed a marked threshold nonlinearity. A train of brief cone depolarizations, first to –40 and then to –35 mV produced a consecutive series of cone tpscs with discrete levels (Fig. 3a, upper panel). Of the 25 trials that resulted in a cone tpsc, only the largest tpsc, corresponding to a content of at least 7 quanta, resulted in a cb1a cell epsc (Fig. 3a, lower panel, thick line). The initial portion of the scatterplot (Fig. 3b, lower panel), within a cone-linear range of 12 vesicles (Supplementary Fig. 4a shows the entire plot), was fitted with a power-law curve that had an exponent of 2.11 (b = 3.00 ± 0.41, mean±S.E., *n* = 10 cone to cb1 cell pairs; see Supplementary Fig. 4b–d for additional examples). In this instance, over the cone linear range, a power-law curve provided a significantly better fit than a line (*p* = 0.0438; F test).

We used a second approach to characterize the response nonlinearity that allowed us to add two cone-cb1a cell pairs to the sample where the deviation of the cb1a cell response from baseline was negligible over the entire cone-linear range (e.g., Supplementary Fig. 4b). By assuming, against the idea of a nonlinearity, that a shallow slope might result from a single dendritic contact linearly sampling release at a single cone invagination, we fitted the scatterplot with a line over a cone tpsc range equivalent to 7 quanta (Fig. 3b, lower panel; Supplementary Fig. 4b; solid blue lines). From the slope, we calculated the cb1a cell epsc current increment (*e.g*., 0.35 pA in Fig. 3b) per cone tpsc event (25.1 pA), and then extrapolated how many epsc increments would be required to produce the maximal cb1a cell epsc response if summation were linear (symbolized by the dashed blue line and arrow). Applying this approach, we calculated that the maximal epsc for the cone to cb1a cell pair in Fig. 3b contained at least 585 quanta (median=853 quanta per maximal epsc; range=167 to 5568; *n* = 12). Thus, the assumption of linearity leads to a contradictory result insofar as a depolarization for 1 ms stimulated a ~16 fF increase in membrane capacitance, equivalent to ~19 vesicles from an individual ribbon or ~370 vesicles from the entire terminal (Fig. 2a), which is considerably less than the median values above. Both the power-law fit and linear extrapolation approaches support the idea that cb1 cell responses increase non-linearly with the amount of cone transmitter released.

Cb3 cell scatterplots also displayed an initial nonlinearly. A scatterplot of cb3a cell epsc versus cone tpsc response (Fig. 3b, upper panel; Supplementary Fig. 5a shows the entire plot) was fitted with a power-law curve that had an exponent of 1.85 (power-law significantly better than linear fit, *p* < 0.0001; b = 2.25 ± 0.10; *n* = 21 pairs, F test; b significantly smaller than that for cb1 cells, *p* = 0.0239, two-tailed unpaired t test; see Supplementary Fig. 5b-e for additional examples). Interestingly, the initial nonlinearity was accompanied by an increase in effective BC unitary event size (calculated as $\sigma^{2}/{\bar{x}}$, 2.33 ± 0.23-fold, mean±S.E., n = 6 cone to cb3 cell pairs; p = 0.0022 different from one, two-sided one sample *t* test) without a change in effective cone event size (1.0 ± 0.2-fold; Supplementary Fig. 5f,g). This observation suggested that an increase in the percentage of multi-quantal events was not responsible for the nonlinearity insofar as multi-quantal events should similarly affect mean-variance calculations for both the cone and BC.

The sampling properties of cb3 and cb1 cell dendrites were further characterized in response failure plots. The postsynaptic cb3a BC whose data is plotted in Fig. 3b responded ~30% of the time when the presynaptic cone released a single quantum of transmitter (Fig. 3d). Two additional cb3 cells had a similar percent response at the single quantal level, while two cb3 cells in the sample did not respond at this level (Fig. 3e). Reponses transitioned to a 90-100% success rate when cone tpscs contained 3–5 vesicles (4 out of 5 pairs). Plots of percent response failure versus cone quanta released were poorly fitted by the model used for cb2 cells (Fig. 3d, magenta curve). Rather, response plots were fitted by a model in which a “success” depended on the exposure of any one cb3 cell cone contact to two or more overlapping release events from up to three neighboring active zones (Fig. 3c). This type of model could fit the rapid transition from a complete response failure when one vesicle was released by a cone to no failures when 3-5 vesicles were released (Fig. 3e, black curve). To account for the observed rate of failures at the single vesicle level, the model could be adjusted so that a variable percentage (e.g., 30%) of individual vesicles elicited a BC response (Fig. 3d, black curve; Fig. 3e, cyan and blue curves). The model suggested that cb3 BCs sample release from nearly all of a cone’s invaginations with a cooperativity of 2 or more, except for occasional responses at the single vesicle level. A similar analysis performed on cb1a cells showed a 90-100% failure rate when the cone released 1–4 vesicles, transitioning to 0% when 11 or more quanta were released (Fig. 3g). Applying the same model (Fig. 3c), cb1a cell responses required the local overlap of 3–5 events at a contact while sampling release from between one-half and nearly all of a cone’s release sites. The insensitivity to transmitter release combined with relatively broad sampling of release sites may be viewed in the context of the cb1a cell dendrite, which contacts the cone terminal in the center of its pedicle cap (Supplementary Fig. 4f–j).

STED microscopic images show that the GluK1-labeled processes of cb3a/b cells form a dense plexus beneath the cone terminal (Fig. 1o). To quantitate the density, GluK1 labeling in a collapsed stack was binarized and individual terminal profiles were superimposed and averaged to calculate the mean label density at a terminal (Fig. 3f, top; Supplementary Fig. 6a, b). Smoothed profiles (Fig. 3f, bottom) consisted of a circular or elliptical ring and central dimple with average densities of 71.1 ± 9.3 and 63.0 ± 13.2% (mean ± S.D., *n* = 5 retinas; Supplementary Table 1), respectively. On the idea that the processes of two GluK1 receptor subunit expressing cb3 cells (i.e., cb3a and cb3b), on average^9^, are distributed throughout the elliptical region (Supplementary Fig. 6c, d; Supplementary Table 2), the uniform, high labeling density supports the view that the dendrites of an individual cb3 cell are well-positioned to access the transmitter released from most cone active zones.

### Simultaneous epscs in Off BCs

**Fig. 4:**
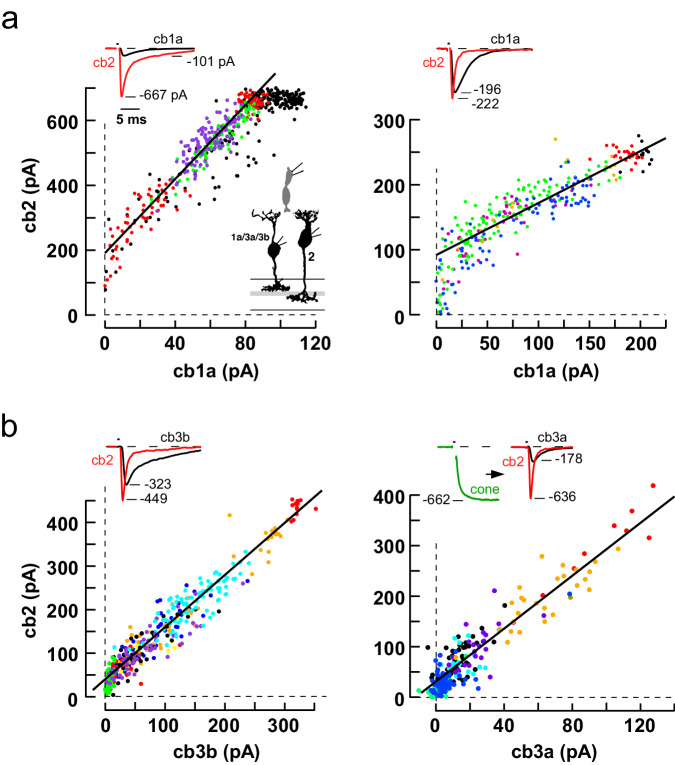
Simultaneous epscs in two BCs postsynaptic to a single stimulated cone. **a** Scatterplots of peak cb2 versus peak cb1a epsc response during several stimulus epochs (different colored points; left, linear fit slope = 5.7 and y-intercept = 190 pA; right, 0.8 and 90 pA). **b** Scatterplot of simultaneous responses in cb2 cells paired with either a cb3b or a cb3a cell (left, slope = 1.2 and y-intercept = 40 pA; right, 2.6 and 31 pA). Insets. Simultaneous peak BC responses (first three plots) evoked by stimulating a cone (1 ms pulse) in the loose seal mode. In the plot on the lower right, cone voltage was maintained in voltage clamp. BC silhouettes republished from Light, A. C. et al. Organizational motifs for ground squirrel cone bipolar cells. J Comp Neurol 520, 2864-2887 (2012). Cone silhouette republished from Neuron, Vol 91, Grabner, C. P. et al. Mechanism of High-Frequency Signaling at a Depressing Ribbon Synapse, 133-145, (2016), with permission from Elsevier.

In cone to cb1a cell scatterplots, the synaptic response nonlinearity frequently overlapped with the onset of cone transporter saturation. To better visualize the nonlinearity, we took advantage of the linear relationship between cone transmitter release and the cb2 epsc response, which we assume extends beyond the transporter saturation point. We evoked transmitter release from a presynaptic cone and compared the simultaneous responses in postsynaptic cb1a and cb2 cells (Fig. 4a). Scatterplots had at least two regions. At low cone stimulus strengths, cb2 cells responded but cb1a cells did not; at medium stimulus strengths, the responses of both cells were linearly related; during the strongest pulses, cb2 cell responses may saturate^18^. Fits to the linear portions of the plots in Fig. 4a had y-intercepts equivalent to 28 (left) and 9 (right) cb2 cell quanta, the former value supporting the idea that >1 released vesicle per ribbon is required for a cb1a cell response. For comparison, scatterplots of responses from cb3b and cb3a cells paired with cb2 cells (Fig. 4b) displayed linear relationships with smaller y-intercepts (~3 quanta). While cb1a and cb2 cells could theoretically sample from different, specialized sets of active zones, correlated fluctuations in the trial-to-trial responses of the postsynaptic pairs during an epoch (Supplementary Fig. 7) imply that the BC types sample release from overlapping active zones. The results from paired BC recordings support a substantial threshold nonlinearity in cb1a cells and a lesser nonlinearity in cb3 cells.

### Contribution of receptor affinity to threshold

**Fig. 5:**
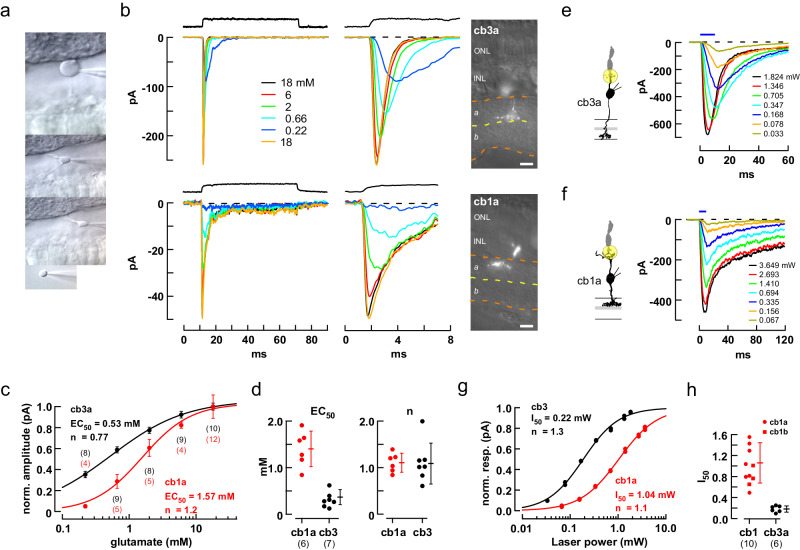
Responses of isolated cb3 and cb1 cell KARs to different concentrations of glutamate. **a** Sequence illustrates the procedure for withdrawing a BC soma while leaving an intact axon and dendrite in the slice. **b** Responses of representative somas to rapid applications of glutamate (0.22–18 mM). Solution exchange temporal profiles (black traces, above). The entire step response is shown on a slow time base (left) and the peak response on a faster time base (middle). Fluorescence images of the remaining axon/dendrite allow identification of the cell type (right; scale bar = 10 µm). **c** Plot of normalized peak response versus concentration for the cells in (**b**). Individual points are averages at a concentration (number of trials shown above and below; mean±S.D.). **d** Categorical scatterplots of EC_50_ and ‘n’ obtained from the Hill fits for cb1a and cb3 cells showing mean±S.D. and number of independent cells in parentheses. Hill coefficients were not statistically different (cb3: 1.09 ± 0.17; cb1: 1.11 ± 0.08; *p* = 0.922; two-tailed unpaired *t* test). **e**, **f** Responses of a cb3a and cb1a cell to glutamate uncaging. A 3 µm diameter uncaging spot, illustrated by the yellow circle, was focused on the dendrites of the recorded BC at a cone terminal. The amount of released glutamate was controlled by adjusting flash intensity (mW; flash duration shown above). 20-80% rise times were 1.23 and 2.17 ms for the maximal cb3a and cb1a responses, respectively. **g** Plots of normalized peak response versus spot intensity for the cells in *e* and *f*. Responses were fitted with a Hill Equation to obtain the laser intensity half-maximum, I_50_. **h** Plots of I_50_ for individual cb1 and cb3 cells. AMPA receptors were blocked with 35 µM GYKI 53655 in all experiments. Cb3a cell I_50_ = 0.18 ± 0.02 mW (mean ± S.E.; *n* = 6 independently recorded bipolar cells); cb1a cell I_50_ = 1.06 ± 0.17 mW; (*n* = 7 independent recordings; significantly different, *p* = 0.0002; two-tailed unpaired *t* test). Cb1a (*n* = 7) and cb1b (I_50_ = 0.98 ± 0.11 mW; *n* = 3) cell I_50_ values were not significantly different (*p* = 0.5082, two-tailed unpaired *t* test). BC silhouettes republished from Light, A. C. et al. Organizational motifs for ground squirrel cone bipolar cells. J Comp Neurol 520, 2864-2887 (2012). Cone silhouette republished from Neuron, Vol 91, Grabner, C. P. et al. Mechanism of High-Frequency Signaling at a Depressing Ribbon Synapse, 133-145, (2016), with permission from Elsevier.

Consistent differences in diffusion distance between release sites and dendritic contacts may affect the gain of a postsynaptic response but should not, on their own, create a threshold. To determine the basis for the threshold in cb1 cells, we compared the concentration-response properties of cb1 and cb3 cell KARs in nucleated patches (Fig. 5a). In 18 mM glutamate, cb3 and cb1a somas had peak response amplitudes of 250.6 ± 73.4 (mean ± S.D., *n* = 7 independent somas) and 40.9 ± 20.6 pA (*n* = 6 somas), and 20–80% rise times of 0.24 ± 0.14 and 0.19 ± 0.06 ms, respectively. Individual cb1a and cb3a cell receptors (Fig. 5b, c) had EC_50_s of 1.57 mM (1.40 ± 0.16 mM, *n* = 6 somas) and 0.53 mM (0.37 ± 0.06 mM; *n* = 7 somas), respectively, for an overall 3.82-fold difference (Fig. 5d; *p* < 0.0001). Average Hill coefficients were close to 1 for both receptors. In a separate set of experiments, we also found a 5-10-fold difference in receptor sensitivity between cb1a and cb3 cells at concentrations between 12.5 and 100 µM (Supplementary Fig. 8a–d), a range that encompasses the estimated concentration of glutamate at cone basal contacts following single vesicle fusion in the absence of glutamate transporters (see below). Receptors also differed with respect to IC_50_ (3.66-fold higher in cb1a cells; Supplementary Fig. 8e–h) and recovery from desensitization (faster in cb1a cells; Supplementary Fig. 8i, j), consistent with a previous report^48^. We addressed the possibility that dendritic and somatic KARs might have different EC_50_s by using a laser spot to uncage MNI-glutamate at the synapse and found that cb1a cell receptors were 5.88-fold less sensitive than those in cb3a cells (Fig. 5e–h). The results suggest that differences in receptor affinity can play a role in the different synaptic responses of cb1 and cb3 BCs, but Hill coefficients close to 1 argue against a role for subunit cooperativity in producing response nonlinearities.

### Contribution of basal glutamate binding sites

**Fig. 6:**
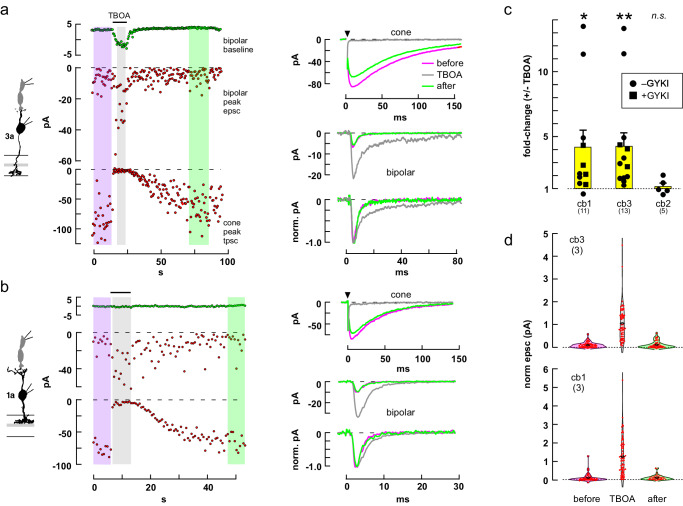
The glutamate transporter blocker TBOA increased small epsc amplitude in cb1 and cb3 BCs. **a** Plots of cb3a BC baseline current (left, top), epsc amplitude (middle), and peak cone transporter current (bottom) as a function of time during a train of 1 ms steps to –30 mV. Average responses (right) before, during, and after puffer application of TBOA. Color coding on the right corresponds to the shaded regions on the left. **b** Similar results for a cone to cb1a cell pair. **c** Aggregate results (different from no change, *p = 0.0324, **p = 0.0094; n.s., *p* = 0.5972; two-tailed one sample *t* test). Experiments on cb1 and cb3 cells were performed both with and without the addition of GYKI53655 (*p* = 0.3843 and 0.7863, no difference, respectively; two-tailed unpaired *t* test). Results in *a* were obtained without and in *b* with GYKI53655. **d** TBOA reduces the number of response failures during a pulse train. Individual epsc peak response amplitudes for 6 cone to bipolar cell pairs (3 cb1 and 3 cb3), before, during, and after TBOA application, were each normalized to the average response in TBOA (center black diamonds) and combined. BC silhouettes republished from Light, A. C. et al. Organizational motifs for ground squirrel cone bipolar cells. J Comp Neurol 520, 2864-2887 (2012). Cone silhouette republished from Neuron, Vol 91, Grabner, C. P. et al. Mechanism of High-Frequency Signaling at a Depressing Ribbon Synapse, 133-145, (2016), with permission from Elsevier.

We next considered whether glutamate transporters at the cone terminal might function as saturable binding sites that reduce the amount of transmitter that can reach cb1 and cb3 cell contacts^49–52^. We tested for a role of transporters in glutamate binding and response attenuation by applying the competitive antagonist TBOA and measuring the change in epsc amplitude and time course. In a cb3a BC (Fig. 6a), TBOA blocked the cone response while producing a 3.48-fold increase in epsc amplitude (4.26 ± 1.05-fold, *n* = 13 independent cone to cb3 BC pairs; Fig. 6c), a 2.80-fold prolongation of response decay (increase in τ_decay_ = 2.39 ± 0.16-fold, *n* = 11; *p* < 0.0001), and an -7.56 pA increase in BC baseline current (-7.70 ± 2.00 pA, *n* = 13; *p* = 0.0023). TBOA also enhanced the epscs in a cb1a cell by 3.53-fold (Fig. 6b; 4.21 ± 1.29-fold, n = 11 independent cone to cb1a BC pairs, Fig. 6c), prolonged response decay by 1.44-fold (1.49 ± 0.94-fold, *n* = 10; *p* = 0.0005), and increased baseline current by -0.38 pA (-9.02 ± 3.46 pA, n = 11; p = 0.0263). Epsc increases were observed under control conditions that allowed recordings at cone to cb2 cell synapses, which demonstrated no change in epsc amplitude (Fig. 6c), and in GYKI53655, which prevented horizontal cells from depolarizing in response to elevated concentrations of glutamate and modulating cone transmitter release^53,54^, but which blocked cb2 cell responses^42^. TBOA not only increased the average cb1 and cb3 epsc response, but also markedly increased the percentage of successful responses during trains that were selected for a high percentage of apparent response failures under control conditions (Fig. 6d). The results suggest that glutamate binding by transporters can reduce basal cleft glutamate at low concentrations resulting in an attenuation or elimination of postsynaptic responses to individual release events.

**Fig. 7:**
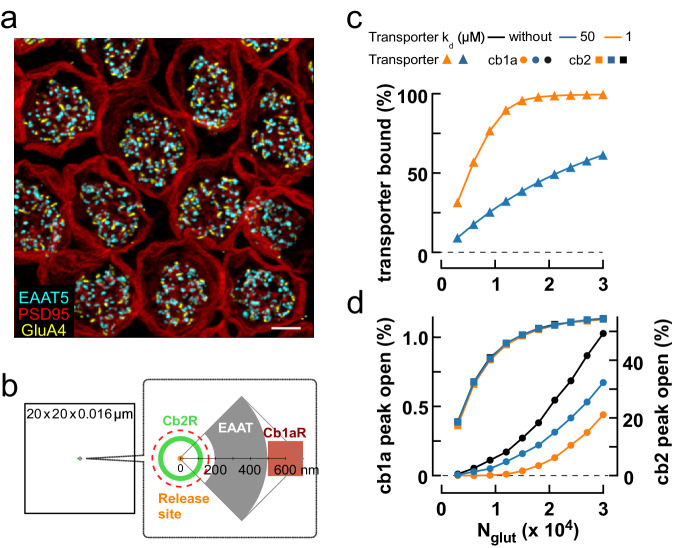
Modeling linear and nonlinear transmission at cone to Off bipolar cell synapses. **a** Collapsed 3D STED image stack of cone terminals immunolabeled for PSD95, a marker for the sides and base of the terminal (red), GluA4 (yellow) which is a marker for invaginations, and EAAT5 (cyan) which is localized to the terminal base but not colocalized with GluA4 labeling (scale bar = 2 µm; see Supplemental Movie 1). There were 62.0 ± 5.4 EAAT5 puncta per cone (mean ± S.D., *n* = 9 cones). **b–d** Monte Carlo simulation of the effect of cone transporters on cb1a and cb2 cell receptor responses to released glutamate. **b** Diagram of the model cleft. **c** Peak transporter glutamate binding as a function of the number of released glutamate molecules (N_glut_) for 3200 transporters in a 0.165 µm^2^ patch (gray area in the diagram). Transporters reversibly bound glutamate with a *K*_*d*_ of 50 µM, a value close to that reported for expressed EAAT5^49^, or 1 µM, similar to the steady state EC_50_ measured by rapid perfusion (Supplementary Fig. 2e, f). **d** Peak channel open probability for an annulus containing 100 cb2 cell AMPA receptors (green in the diagram) or a patch containing 350 cb1a cell receptors (red in the diagram; area = 0.04 µm^2^) in the absence of transporters or with transporter *K*_*d*_’s of 1 or 50 µM. Simulations on each receptor type were performed separately. Red dashed circle delimits the extent of an invagination in this flat-surface model of the synaptic cleft.

To assess the impact of basal transporter localization on Off BC responses, we created a simplified Monte Carlo model of glutamate diffusion in the cone synaptic cleft following vesicle fusion. The model assumed that transporters were localized to patches of basal membrane (Fig. 7b; Supplementary Fig. 9d). An antibody to the glutamate transporter EAAT5 strongly labels mouse cone terminals^50^, and STED images of the ground squirrel terminal show EAAT5 puncta at sites that are both more numerous and displaced from GluA4 puncta, a marker for invaginations, justifying this assumption (Fig. 7a). The glutamate transporter EAAT2 is also localized to the outerplexiform layer^50,51^. We further assumed that transporters act as reversible binding sites on the time scale of an epsc, consistent with the slow transport rate of EAAT5^49^. Modeled cb1a cell receptor responses replicated actual responses with respect to response time course, EC_50_, IC_50_, and recovery from desensitization (Supplementary Fig. 9a–c). However, at low concentrations of glutamate, similar to those predicted to occur at cone basal contacts, model receptor responses displayed a moderate cooperativity (Hill coefficient = 1.73) which differed from the more linear relationship experimentally observed (Supplementary Fig. 8d). In simulations, adding transporter binding sites to a location between the release site and cb1a cell postsynaptic receptors reduced the amount of glutamate that reached receptors and enhanced the initial response nonlinearity in direct relation to transporter glutamate affinity (Fig. 7c, d). At higher glutamate concentrations, transporter binding sites saturated and the effect on receptor response was reduced as signified by the upward curvature of the cb1a cell receptor response and its subsequent parallel rise relative to control. Adding transporters to the opposite side of the release site relative to receptors or beyond the receptor patch (*e.g*., on distant Muller cell processes) had little effect on peak epsc responses, analogous to the case in which basal transporters did not affect the linear rise of invaginating cb2 cell receptor responses. The model provides additional support for the idea that saturable sites on glutamate transporters can buffer glutamate at low levels of transmitter release, reducing the activation of receptors on postsynaptic cb1 cells.

### Spatiotemporal glutamate gradient at the cone terminal

**Fig. 8:**
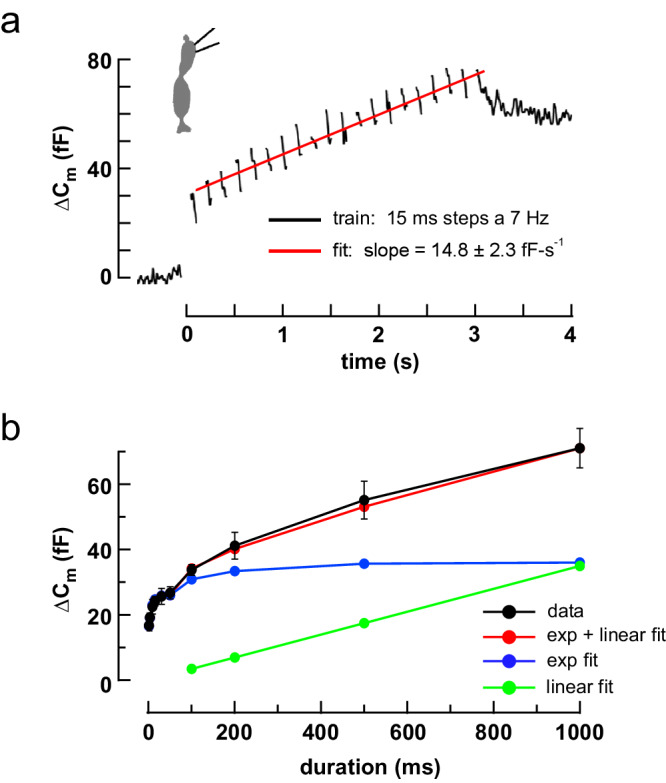
Maximal steady rate of vesicle replenishment at the cone terminal. **a** ΔC_m_ during a train of 20 × 15 ms steps from -70 to -10 mV at 7 Hz. Average of 8 cells. Intercept = 30.6 fF. **b** Plot of average ΔC_m_ versus step duration (step to –10 mV; black circles and straight lines; 9 cells; mean ± S.E.). The plot was approximated by summing two components, a bi-exponential fit to the rise at early times (blue circles and lines) and a linear fit to the ΔC_m_ values between 0.2 and 1.0 s (green points and line). The steady replenishment rate was obtained from the slope of the green line (37.6 ± 3.7 fF-s^–1^). Cone silhouette republished from Neuron, Vol 91, Grabner, C. P. et al. Mechanism of High-Frequency Signaling at a Depressing Ribbon Synapse, 133-145, (2016), with permission from Elsevier.

Cone photoreceptors maintain a steady rate of vesicle fusion when depolarized in the dark. If the rate is low, fusion events at the ~20 ribbons in a cone and their associated glutamate gradients will effectively be spatiotemporally discrete, each resembling those evoked by a weak 1 ms depolarization. In this case, the properties of the steady response can then be inferred from those of the small, evoked responses. We estimated the maximal steady rate of vesicle turnover by using two approaches. The first approach (Fig. 8a) yielded a lower estimate, ~17 vesicles-ribbon^–1^-s^–1^, by monitoring the change in membrane capacitance during a train of depolarizing pulses with high intracellular EGTA (10 mM)^39,55–58^. The second approach used depolarizing steps of up to 1 s in length and established a rate of refilling under continual Ca^2+^ entry equivalent to 44 vesicles-ribbon^–1^-s^–1^ (Fig. 8b). The results suggest that continuous Ca^2+^ entry accelerates turnover by 2.6-fold in agreement with previous studies^58^. We adopted a middle-value replenishment rate of 30 vesicles-ribbon^–1^-s^–1^ or ~600 vesicles-s^–1^ per terminal. A model of glutamate diffusion in the cleft shows that an individual event raises local basal glutamate concentrations above 10 µM for a very short interval (<1 ms; Supplementary Fig. 9d, e). During steady release, where one vesicle fuses at a ribbon on average every 33 ms, a simple calculation suggests that fewer than 10% of the event profiles from the same and adjacent ribbons will significantly overlap in space and time. Taken together, the results predict that cb1a cells will respond less than cb2 or cb3 cells under steady, uni-vesicular release conditions, responding instead when a cone depolarization triggers near coincident fusion at the same or adjacent ribbons.

### Threshold signaling during light responses

**Fig. 9:**
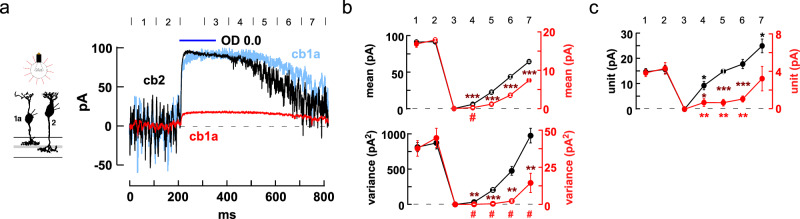
Threshold nonlinearities in cb1 cell responses during light increments. **a** Responses of individual cb2 (black trace) and cb1a (red trace, original; light blue trace, scaled) cells simultaneously recorded during a 150 ms step of OD 0.0 light (1.575 × 10^7^ equivalent photons-µm^–2^-s^–1^). Epochs (duration = 100 ms) are marked by numbers (above). Average of 4 trials. **b** Mean current (above; obtained by averaging the average currents from corresponding epochs across the trials; mean ± S.E. for all plots) and variance (below; obtained by averaging the variance of each of the corresponding epochs; red and black symbols and axes correspond to cb1a and cb2 cell currents, respectively). Axes were scaled for comparison. Cb1a and cb2 cell plots were normalized based on the average values in the first two epochs corresponding to the initial levels in darkness. Smaller S.E. bars are within plot symbols. Maroon (combination of black and red) asterisks indicate a significant difference between the normalized responses in an epoch (cb1a different from cb2: epochs 4-7, current mean, *p* = 0.0009, 0.0001, 0.0001, 0.0003; current variance, *p* = 0.0017, 0.0001, 0.005, 0.007; two-tailed unpaired *t* test). The absence of an asterisk indicates a n.s. change. The red hash marks indicate that the cb1a responses were not significantly different from zero (mean, *p* = 0.064; variance, *p* = 0.4236, 0.0906, 0.0520, 0.1129; two-tailed one-sample *t* test). **c** Plot of effective unit amplitude versus epoch. Red and black asterisks indicate significantly different from the average values in the dark for the cb1a (*p* = 0.0037, 0.0023, 0.0025, 0.5594) and cb2 (*p* = 0.0394, 0.7668, 0.2429, 0.0367; two-tailed one sample *t* test) cell, respectively. When effective unit sizes for both cells were normalized to their preceding values in the dark, the unit size in the cb1a cell recovered more slowly after a flash (maroon asterisks, cb1a versus cb2, *p* = 0.0255, 0.0002, 0.0007, 0.0584; two-tailed unpaired *t* test). Effective unit amplitude was calculated within a trial epoch and then averaged across the 4 corresponding epochs. BC silhouettes republished from Light, A. C. et al. Organizational motifs for ground squirrel cone bipolar cells. J Comp Neurol 520, 2864-2887 (2012).

Differences in the threshold responses of the Off BC types should be most evident in bright light where cones are hyperpolarized and vesicle fusion rates are low. We examined BC light responses in two types of experiments. In the first type of experiment, we applied 150 ms steps of bright light that produced a saturating hyperpolarized response in cones and then simultaneously monitored synaptic currents in cb1a and cb2 cells as cone membrane voltage slowly recovered to its depolarized dark state^59^. In the dark, both BCs had fluctuating inward currents due to continuous glutamate release from cones. A bright light stimulus suppressed cone transmitter release reducing both inward current and synaptic fluctuations. During the slow return to baseline, plots of BC current mean and variance (Fig. 9b), both measures of synaptic activity, showed a faster recovery in the cb2 than in the cb1a cell.

Campbell’s Theorem (Eq. (1)) relates the ratio of continuous signal variance and mean to the average amplitude of component unitary events when event time course is known^60^. Cb1a and cb3 cell unitary event time courses can be obtained from small evoked epscs or spontaneous events^35^. Effective unitary event amplitudes were calculated during epochs prior to (1-2) and after (4-7) the stimulus (Fig. 9c). For the cb2 cell, the effective unit size at the start of the recovery (epoch 4) was reduced by 38.8 ± 11.1% relative to the size in the dark prior to the flash, transitioning to a 64.1 ± 17.8% increase in epoch 7. In the cb1a cell, unitary responses were decreased by 82.8 ± 10.0% during epoch 4 returning to the original level by epoch 7. The reduction in normalized effective event size was significantly greater in the cb1a than in the cb2 cell during each phase of the recovery, consistent with an approach to threshold in cb1a cells.

**Fig. 10:**
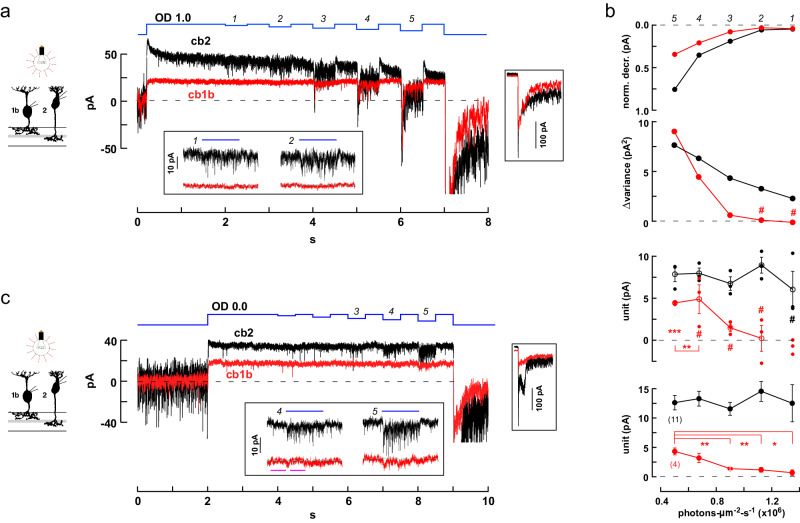
Threshold nonlinearities in cb1 cells during light decrements. **a** Responses of simultaneously recorded cb1b and cb2 cells during a long step of OD 1.0 light that was interrupted by a series of decrements (average of 3 traces; stimulus sequence in blue). Lower inset. Magnified view of the light responses during the first and second decrements. Right inset. Responses at light-off shown on the same time scale but with a compressed current axis. **b** Above. The normalized change in membrane current was plotted against the light intensity during the decrement (i.e., the ‘step-to’ light intensity with the result that larger decrements are plotted toward the left). Current was normalized by setting the average immediately before a decrement equal to 0 relative to the resting current, 1, in the dark. Normalized steady cb1b and cb2 responses were significantly different during all decrements (mean ± S.E.). Upper middle. Change in response variance, before versus during a decrement, is plotted against ‘step-to’ light intensity. Hash marks denote either negative change or no change (Levene’s Mean Statistic). Lower middle. Effective unit amplitude was calculated by separately analyzing each of the 3 trials and plotted against light ‘step-to’ intensity (open circles are mean ± S.E. while closed circles show individual data points). Hash marks denote n.s. difference from 0 (two-tailed one sample *t* test). Below. Aggregate unitary responses for 11 cb2 and 4 cb1a/b BCs independently recorded. Asterisks indicate that the values of the cb1 cell unit were significantly reduced during decrements 1–3 relative to decrement 5. See Statistics and Reproducibility for additional *p* values and criteria. **c** Decrement responses from a different cb1b and cb2 cell pair during a long OD 0.0 background step (average of 2; repetitions were limited by photopigment bleaching). The cb2 cell response displayed prominent epscs in the absence of events in the cb1b cell (decrements 3, and lower inset decrements 4 and 5). Magenta bars indicate the before and during regions used to measure the activity change in the cb1b cell in response to decrement 4 (200 ms intervals; *p* = 0.1930; not significantly different, two-tailed unpaired *t* test). BC silhouettes republished from Light, A. C. et al. Organizational motifs for ground squirrel cone bipolar cells. J Comp Neurol 520, 2864-2887 (2012).

In the second type of experiment, we applied a steady bright light to suppress cone release and, after a 2 s interval to allow cb1 and cb2 receptors to recover from desensitization, applied a series of stimulus decrements to slightly increase transmitter release. In a simultaneous recording from nearby cb2 and cb1b cells (Fig. 10a), visual inspection suggests that the cb2 cell responded during decrements 1 and 2, while the corresponding responses in the cb1b cell were minimal or absent (Fig. 10a, inset). More quantitatively, when cb1b and cb2 cell responses during a light decrement were normalized with respect to the steady current in darkness (Fig. 10b, above), the cb2 cell response was significantly larger than the cb1b response during each decrement. Although statistically significant, the response amplitudes of the cb1b cell during the first two decrements were small and lacked an increase in variance (Fig. 10b, upper middle) which normally accompanies a response to vesicular transmitter release. Effective unitary responses were calculated by comparing the mean change in current amplitude and variance during a decrement (Fig. 10b, lower middle). The cb2 cell’s unitary responses were roughly constant at ~7-8 pA for all decrements. In comparison, the cb1b unit could not be calculated during the first decrement due to a small reduction in variance. The calculated unit size was not different from zero during individual increments 2–4, although with an increasing trend, and attained significance during decrement 5 (~5 pA, *p* = 0.0005, different from 0, two-tailed one sample *t* test) and when the data points from decrements 4 and 5, which had similar mean values, were combined (*p* = 0.0017). Plots of unitary event amplitude versus light decrement intensity (Fig. 10b, below) were also obtained by averaging data from 2 paired and 11 individual recordings performed at approximately the same background intensity. The effective cb2 unit remained relatively constant in relation to decrement size whereas the cb1 cell unit was larger during decrement 5 and significantly reduced during smaller light decrements. Finally, an additional paired recording was performed during a very bright steady light background that fully blocked synaptic activity in both a cb2 and cb1b cell (Fig. 10c). We observed vigorous epsc activity in the cb2 cell during decrement 4 contemporaneous with an absence of activity change in the cb1b cell. The results are consistent with the prediction that bright light reduces or eliminates event noise in cb1 cells under conditions where signaling persists in cb2 cells.

## Discussion

The combination of basal and invaginating contacts is unique to the cone photoreceptor synapse, but the functions of these ultrastructural features are poorly understood. Here we have shown how differences in dendritic contact location relative to release sites, contact numbers, glutamate transporter localization, and postsynaptic receptor properties can sensitize the Off bipolar cell types to different features in the cone vesicle stream. Cb2 cells make multiple invaginating contacts close to ribbon release sites and sum events linearly starting at the single event level. Cb3 dendrites make multiple contacts that are distant from ribbon sites and fail to respond to most single events, instead responding to two or more locally coincident events resulting in a modest nonlinearity. Remarkably, for cb1 cells, which make a single or small numbers of tiny contacts in the center of the cup-like base of the cone terminal, the individual transmitter quantum is not the fundamental unit of neurotransmission. Instead, cb1 cells exclusively respond to coincident multivesicular or multiquantal events resulting in a distinct nonlinearity.

While vertebrate retinas share a general plan, specific retinal neuron types, even among mammals, display adaptations to ecological niche^61^. Ground squirrel cones have the fastest light responses among mammals^62^, and flicker ERG responses also suggest that post-receptoral processing is fast^63^. The glutamate receptors on invaginating cb2 cells are closer to release sites than those of other mammalian Off BC types which instead make basal contacts. However, the dendritic tips of invaginating cone BCs are still separated from ribbon active zones by 80–120 nm^30,37^. Hence, it was important to demonstrate as we did that cb2 cells epscs are not only fast^35^ but result from the summed responses to individual quanta. Supplementary Figure 10 shows simulated cb2 cell light responses based on a linear summation response rule. Horizontal cells, whose processes are even closer to ribbon release sites in invaginations, also respond linearly to rod transmitter release^64^.

For cb3 cells, the paradox of a minimal response following single vesicle fusion and a reliable response following the fusion of 3–5 vesicles can be resolved if cb3 cell dendrites, as a group, sample release from nearly all of a cone’s invaginations (Supplementary Fig. 10). Complete sampling is supported by STED microscopic images that show an extensive network of GluK1 labeling beneath the terminal (Supplementary Fig. 6) and the observation that cb3 cell receptors can respond to glutamate near the concentrations found at basal sites (Supplementary Fig. 8a, d). Responses near the quantal level may be reduced by transporter glutamate binding, a saturable effect (Supplementary Fig. 2d-f) that could account for the decrease in effective unit size during small epscs (Supplementary Fig. 5f, g). An event amplitude that scales with release rate may reduce the impact of quantal noise on signaling during bright light, where the rate of vesicle release is low and small decrements in light intensity produce small responses that are optimally encoded by small events. In addition, the results support an optimization that has previously been described only at fly photoreceptor synapses^65,66^. This optimization is based on the idea that while the mean rate of release at each of a cone’s ribbons is governed by membrane voltage, the timing of release is stochastic and independent of release at neighboring ribbons. Therefore, a BC can improve its estimate of the cone signal by sampling from multiple ribbons up to the limit of the entire terminal population. Cb3 as well as cb2 cells may take advantage of this noise reduction strategy.

A threshold nonlinearity at cone to cb1a cell synapses is directly supported by the positive y-intercept in scatterplots of cb2 versus cb1a cell epsc response (Fig. 4a). Differences in sensitivity to small decrements on a bright background (Figs. 9–10) show that the cb1a cell nonlinearity affects light responses. A threshold offset is challenging to model insofar as the concentration gradient of a diffusing transmitter should scale with the total amount released irrespective of the distance between release site and receptor cluster. An alternative possibility, that cb1a cell receptors have a large Hill coefficient, is contradicted by the results. Rather, modeling suggests that the offset is caused by a combination of diffusion distance (Fig. 1f-j; Supplementary Fig. 4g–j), glutamate binding at basally located transporters (Figs. 6–7), and a cb1a cell receptor with a high EC_50_ (Fig. 5b–d). During moderate to strong cone depolarizations, we found that the epsc amplitudes of the Off BC types are linearly related^4^. We hypothesize that the linear relationship occurs under conditions where release saturates glutamate transporter binding sites and cb1a, cb2, cb3 cell receptors respond to incremental increases in glutamate concentration with Hill coefficients of ~1. A nonlinearity is also found at some cone to Off BC synapses in the salamander, but the mechanism is unclear^67^.

Threshold filtering reduces the responses of both cb1 and cb3 cells to individual quanta, but both cell types can respond to multivesicular or multi-quantal events. In multivesicular release, overlapping glutamate concentration profiles result from the independent fusion of nearby vesicles whose release is synchronized by a rapid depolarization, a condition related in cones to the depth of a light-dark transition and time in the preceding light. The results suggest that cb1a cells preferentially signal these light decrements while minimizing the transmission of stochastic quantal noise during steady illumination (Figs. 9–10; Supplementary Fig. 10). Multiquantal events are thought to result from either the simultaneous fusion of ribbon-adjacent vesicles^28^ or from compound fusion^26,68^. The threshold in cb1 cells raises the possibility of a multiplexed code at the cone synapse where uni- and multi-quantal events are preferentially signaled by different Off BC pathways. Multiquantal release extends the dynamic range of BC synapses in zebrafish^69^ and is regulated by presynaptic protein composition^64^ and dopamine^70^.

The different properties of cb1a/b and cb3a/b cells may have analogs in other mammalian retinas. Results from primate and mouse support the idea that Off BCs use two different types of KARs. In the primate, one set of Off BCs, consisting of the DB2 and DB3b, has GluK1 receptors with properties like those of cb3a and cb3b cells^71–74^. Of the remaining types, the receptors on FMB and DB1 cells most closely resemble those of ground squirrel cb1a/b cells^73^. A similar dichotomy is found between types 3b and 4 in the mouse, which express GluK1-containing KARs, and types 1 and 2, which do not^75,76^. Thus, the strategy of distributing signals to basally-contacting Off BC types with different receptor properties may apply to the primate and mouse.

Our results show that the Off BC types sample the output of the cone terminal with different tradeoffs. The invaginating cb2 cell trades a reduced dynamic range for sensitivity and speed of signaling^18^. Cb1a cells trade sensitivity to local changes in glutamate concentration for the ability to encode the higher, global changes in concentration that result from strong visual stimuli. Cb3 cells sample from almost the entire population of ribbons, providing a high-fidelity response to the cone signal over a broad range, but lack sensitivity to quantal release. The design of the cone synapse enhances coding efficiency by sorting different components of the graded cone signal into more specialized, parallel Off bipolar cell pathways^77^.

## Methods

### Preparation and electrophysiology

All procedures were performed at Northwestern University and approved by the Institutional Animal Care and Use Committee. Retinas were obtained from both male and female thirteen-lined ground squirrels (*Ictidomys tridecemlineatus*) in approximately equal numbers. Retinal slices, 100 µm thick, were obtained using a razor mounted on vertical slide from ~2 × 2 mm squares mounted vitreal side down on Millipore filter paper^35,78^. For experiments involving light responses, retinal slices were obtained under dim red illumination^79^. During recordings, slices were visualized with a Zeiss Axioskop-2FS microscope using a 63x water immersion objective under infra-red illumination. Recordings were made with Axopatch 200B amplifiers (Molecular Devices) and signals were filtered at 5 kHz and digitized at a rate of 10 or 16.6 kHz with a HEKA ITC-18 A/D board (HEKA Elektronik) operated with custom software (Igor Pro 6.21; WaveMetrics). Patch pipettes were pulled from borosilicate glass capillary tubes to tip resistances of 8-12 MΩ.

The external solution consisted of (in mM): NaCl 115, KCl 3.1, MgSO_4_ 2.48, glucose 6, Na-succinate 1, Na-malate 1, Na-lactate 1, Na-pyruvate 1, CaCl_2_ 2, and NaHCO_3_ 25, 0.05% phenol red, and was equilibrated with 5% CO_2_/95% O_2_ to a pH of 7.4. Picrotoxin (50 µM; Sigma, P1675) and strychnine (10 µM; Sigma, S0532) were included in the bath. The patch pipette solution for the BC contained (in mM): KCl 120, K_3_-EGTA 10, MgSO_4_ 2, HEPES 10, ATP 5 and GTP 0.5; pH 7.35 with KOH. The cone pipette solution contained (in mM): KSCN 115, K_3_-EGTA 10, MgSO_4_ 2, HEPES 20, ATP 5 and GTP 0.5; pH 7.35 with KOH. Intracellular solutions contained combinations of a non-fixable tracer, either Sulforhodamine 101 (Molecular Probes, S359) or BODIPY 492/515 (Molecular Probes, D3238) and a fixable tracer, either Cascade blue hydrazide, trilithium salt (0.1 mM; Invitrogen, C3239) or 0.5% Neurobiotin Tracer (Vector Laboratories, SP-1120). Labeled cells could be visualized using immunohistochemistry followed by confocal imaging^6^ or immediately following recording with a Prime95B (Photometrics) camera controlled by µManager (micro-manager.org). All solutions were corrected to an milliosmolarity of 285 ± 5. Experiments were performed at 32-33 ^o^C. Pharmacological agents include: UBP310 (Tocris, #3621), GYKI53655 HCl (Tocris, #2555), DL-TBOA (Tocris, #1223), dihydrokainic acid (Tocris, #0111), and glutamic acid (Sigma, G1251). Membrane capacitance was measured with a HEKA EPC-10 using the ‘sine+dc’ Lockin routine in the Patchmaster software (2x73.3). For capacitance measurements, CsCl 112 and KCl 8 mM were substituted for KCl in the intracellular solution and solution pH was adjused with CsOH^18^. Retinas were stimulated by a light-emitting diode (574 nm) attached to a microscope video port. LED intensity was controlled by pulse-width modulation. Light sources were calibrated with a photodiode detector (International Light) that was positioned beneath the microscope objective. Light intensity was converted to photons at 520 nm, the λ_max_ for the ground squirrel green cone pigment^80^. Figure legends show light intensities. During light stimuli, unitary response amplitude and number were analyzed using Campbell’s theorem^60^,

1 $$\frac{{\sigma }^{2}}{\bar{x}}=\frac{\int {F}^{2}\left(t\right){dt}}{\int F\left(t\right){dt}}$$

where $\bar{x}$ and ${\sigma }^{2}$ are the signal mean and variance and the integrals are equal to event area and event area squared. Canonical events were obtained from cb1 and cb2 cells during 1 ms cone depolarizations. The event amplitude was scaled to find the unique ratio that matched the variance to mean ratio. Application of Campbell’s theorem assumes both linear event summation and that the predominant source of shot noise doesn’t change with light step intensity. Both conditions may be approximate during BC light responses over a range of intensities. Variance and mean measurements after a strong flash (Fig. 9a) were obtained after subtracting a sloped, linear baseline.

### Acquisition and analysis of tpscs and epscs

Epsc and tpsc traces were Gaussian filtered in software with cutoffs of 1000–1250 Hz and 500-750 Hz, respectively, before further processing. Cones were stepped in voltage clamp from a holding potential of –70 mV to a level between –50 and 0 mV for 1 ms. Slow tpsc responses during an epoch (a train of steps to the same voltage) were measured as the average current during a 5 ms window that encompassed the inward current maximum. Average baseline current in an 8 ms window prior to the step was subtracted from the maximum. Peak responses from one or more consecutive epochs were accumulated into amplitude histograms. Amplitude histograms were fitted by a sum of Gaussians with variables for individual peak amplitudes (including failures), mean quantal size (Δx), and failure and success peak Gaussian widths, σ_f_ and σ_s_, respectively. The peak location of the failure distribution, x_0_, was unconstrained. The Gaussian widths for the failure and subsequent success peaks followed the formula (σ_f_^2^ + *n*(σ_s_^2^))^1/2^ where *n* is peak number. Each tpsc response from an entire run consisting of multiple epochs was assigned an effective quantal content, *m*, by binning according to x_0_ + Δx(*m* - 1/2), x_0_ + Δx(*m* + 1/2). Tpscs with *m* ≤ 12 were assumed to sum linearly and constituted the cone-linear range in scatter plots. Assignments were inspected for the presence of spontaneous cone transporter events either before or immediately after the 1 ms stimulus. In the case of overlap, amplitudes were either assigned manually or, in rare cases where assignment remained uncertain, the trial was discarded.

Epsc peak amplitude was determined for all Off BC types using automated routines^35^. In brief, copies of the “original” traces were filtered in software with a Gaussian filter (cutoff frequency 200 Hz). The baseline noise was calculated from time zero to the start of the 1 ms step for each filtered trace and the values for all individual traces were averaged to obtain σ_n_. The response threshold was empirically set to 3.5σ_n_, which was typically 0.8–1.5 pA. Each 200 Hz filtered trace was then sorted into one of two groups, failures or successes, based on whether the epsc exceeded threshold during the response interval which was determined by taking an average over all traces. Next, to determine the event shape, all original epsc responses in the series were averaged and the result fitted by least squares minimization with a function that could approximate both fast and large cb2 and slow and small cb1/3 events. The shape of the function, ConvExpDiffusion, was determined by convolving a fast exponential rise (τ = 0.1 ms) and decay (τ = 1.0 ms) with a profile obtained from an equation for diffusion from a point source in three dimensions. The values from the diffusion equation for peak amplitude, diffusion radius, and start time were saved. For responses in the ‘success’ group, peak amplitude, start time, and rise time were determined for each original trace by fitting with ConvExpDiffusion. For responses in the ‘failure’ group, original traces were fitted within the average response shape that was scaled by amplitude to produce a minimization of the sum of squares. Amplitudes could be positive or negative. The fitting routine issued error messages for instances of poor fit, which could then be inspected for interference by spontaneous events or noise. The fitting routine was robust to small cb1 and cb3 cell epscs whose rise and peak regions were often interrupted by apparent single channel transitions.

### Calculation of confidence intervals in percent response failure plots

For plots of percent failure versus cone quantal content, the confidence interval for each point was based on a maximum likelihood estimation for coin tosses. For a biased coin with an intrinsic probability of heads, *p*, the binomial distribution gives the probability of observing a specific number of heads, *h*, in *n* trials. The cone to Off BC synapse presents the inverse problem where *h* (i.e., the number of failures) and *n*, the number events with a specific tpsc quantal content, are known, and the task is to estimate the likelihood that the intrinsic probability, *p*, is within a certain range of values. The relative likelihood of an intrinsic probability *p* given *n* and *h* is obtained from the beta probability distribution with parameters *h* + 1 and *n* – *h* + 1 such that the greatest likelihood occurs at *p* = *h*/*n* and the confidence interval is bounded by the probability values that exclude the lower and upper 25% of the area under the curve, respectively (*i.e*., the 50% confidence interval). Sample sizes of 2 or smaller were omitted from plots due to the large uncertainty associated with the measurement.

### Rapid perfusion of somatic patches

A rapid perfusion pipette was mounted on a piezoelectric actuator (Burleigh, PZS-200) and driven by an amplifier (Burleigh, PZ-150 M). The command voltage for the amplifier step, typically 18 or 60 ms in duration, was filtered in software by convolution with a Gaussian (width = 1.2 ms) to damp oscillations. Four-barreled glass (Vitrocom, Mountain Lakes, NJ) was mounted on a Kopf vertical puller and pulled so that each square barrel had a width of ~100 µm. The two side barrels were sealed with Sylgard. Solutions were fed under pressure to the two central barrels via small bore polyimide-coated quartz tubes (Polymicro Technologies, Phoenix, AZ). The flow into one barrel contained control solution and had a single input. The flow into the other barrel could be switched among five test solutions. Control solution contained (in mM): NaCl 125, KCl 3.1, MgSO_4_ 1.24, CaCl_2_ 2, and Na-HEPES 10. pH was adjusted to 7.4 with NaOH and osmolarity was adjusted to 285 ± 5 mosm with NaCl. Na-glutamate (18 mM) was substituted for NaCl to make the stock test solution. Serial glutamate dilutions were made by combining stock and control solutions. Junction potential differences between the control and 18 mM test solutions were used to measure open tip switching time at the end of an experiment.

To withdraw BC somas, whole cell access was obtained with a pipette filled with BC intracellular solution containing sulforhodamine 101 and Neurobiotin Tracer. Continuous negative pressure was applied to the pipette by syringe and the soma was slowly withdrawn from the slice leaving behind a soma-less but otherwise intact cell remnant that could be identified under epifluorescence or, after fixation, by confocal microscopy.

For glutamate uncaging, 5 mM MNI-caged-L-glutamate (Tocris, #1490) was added to the extracellular solution and applied to the synapse by a local puffer pipette. BCs did not respond to puffer-applied caged-glutamate in the absence of the flash. Spots, ~3 µm in diameter at the level of the slice, were delivered by a Vortran Stradus Laser (405 nm, nominal power = 50 mW) via a Rapp OptoElectric Spot Illumination System that was attached to the epifluorescence port. Laser power was set using Vortran Stradus Control Software and an electronic shutter was driven by a TTL pulse from the ITC-18. The microscope objective was an Olympus LUMPlanFLN 60x/1.00 W.

### Modeling vesicle fusion at the cone synapse

#### Cb2 cell model

The model cone terminal contained 20 invaginating transmitter release sites of which *x* were contacted by dendrites. The probability of response failure, *p*_*f*_, was calculated as a function of the number of simultaneously released cone quanta, *n*, for each *x* between 1 and 20. Every quantum had an equal probability of release at all 20 sites. For the case where a success occurs whenever a single vesicle is released at a site that is occupied by a dendrite, the probability of failure *p*_*f*_(*n*) = (1 - *x*/20)^*n*^. Probability curves were calculated for each *x* and associated with a cb2 cell data set by least squares minimization. *p*_*f*_(n) plots at a given *x* consisting of discrete points were fitted with an exponential decay curve for display (e.g., Fig. 2g).

#### Cb1/3 cell model

The model cone terminal had 19 invaginating release sites arranged in a trigonal array (Fig. 3c). Contact number could be varied from 1 to 24 with individual contacts located at the centers of triangles whose vertices were release sites. A response rule was constructed and, for a given number of dendritic contacts, the relationship between the failure probability *p*_*f*_ and the number of released cone quanta, *n*, was determined by running a simulation. In a typical response rule, a success occurs when any one dendritic contact receives 2 or more (or ≥3, ≥4, or ≥5) quanta in any combination from the 3 surrounding vertices. Computationally, in a trial, each bin in an array of 19 release sites is populated with *m* release events where *m* = 0, 1, 2, 3… as determined by the Poisson distribution and a random number generator. The Poisson average in a simulation was set to *n*/19 where *n* is the user assigned total number of quanta released in the trial. To determine the *p*_*f*_ for *n* cone quanta, the program generated 1000 trials while only retaining those where the sum of all released quanta across bins equaled *n*. For example, to determine *p*_*f*_ for the condition in which a cone terminal releases exactly 2 quanta, the sum across all retained bins must equal 2. The retained ‘release site’ trial array was then used to populate a 24-bin “dendrite” trial array using the mapping from Fig. 3c and the response rule. For example, for the ≥2 rule above, dendrite array bin 1 would be set to success (=1) if the sum of release site array bins 2, 3, and 6 is ≥2 or failure (=0) otherwise. An analogous operation is then performed for each of the 24 dendrite bins. For each retained trial, if the sum of the dendrite array is 0, then the trial is a failure, otherwise it is a success. The proportion of failed trials was then calculated to give *p*_*f*_ for the number of cone quanta released. The simulation was repeated for *n* = 1… 20 to obtain the entire probability curve. The role of contact number was examined by masking the dendrite array so that the values in the first *x* bins were maintained while values in the remaining bins were set to 0. To model a certain percentage of success at the single quantal release level while otherwise maintaining the ≥2 response rule, 20-35% of the bins that contained 1 vesicle in the retained array were randomly tagged so as to guarantee a success when mapped to a bin in the dendrite array. To simulate multiquantal release, each of the *m* events in each release array bin was associated with a Ca^2+^ channel opening that could randomly lead to mono-vesicular release 80% of the time and di-vesicular release 20% of the time. The total release events in the bin were adjusted accordingly. For trial retention, the sum of all quanta still had to equal *n*. Hence, multi-quantal release does not contribute to *p*_*f*_ when the model cone releases only a single quantum (*n* = 1). Fits were evaluated by least squares minimization against data points and the best fit was arrived at through an iterative procedure.

### Processing tissue for IHC and STED microscopy

Tissue was either fixed in 4% PFA using standard procedures^6^ or in 2% glyoxal solution^81^. Glyoxal fixative (10 ml) contained 7.325 ml distilled H_2_O, 2 ml absolute ethanol, 0.5 ml glyoxal (40% in water, Sigma-Aldrich, #128456), 0.075 ml glacial acetic acid, and 0.1 ml Na-acetate (3 M, Ambion, #AM9740) to give a pH of ~4.0^81^. Pieces of freshly dissected retina with pigment epithelium, 1 × 1 mm, were placed ganglion cell side down onto dry Millipore filter paper (3.0 µm MCE Membrane, #SSWP02500) in a small petri dish. Cold PBS was added, and the pigment epithelium was peeled from the paper-adherent retina. The petri dish was placed on ice for 5 min and then cold glyoxal solution was substituted for the PBS. After 2 hrs on ice, the tissue was placed in the refrigerator at 4 ^o^C for 2 days. Tissue was removed from the refrigerator and washed 3x for 20 min each with cold DPBS (Gibco, #21600-010), and sliced with a tissue chopper into 100 µm or 300 µm thick sections for cross-sectional or whole mount views, respectively. Individual slices were transferred to MatTek 3 mm glass bottom culture dishes and incubated for 1 day in block solution containing 0.5% Triton X-100, 0.1% Na-azide, and 3% donkey serum in 0.1 mM phosphate buffer. Whole mounts were treated with primary antibodies for 6 days (4 days for slices) in block at 10 ^o^C with gentle shaking. The tissue was then washed 6x for 30 min each and incubated in secondary antibody solution with block for 4 days (2 days for slices) with shaking at 10 ^o^C. The tissue was then washed 6x for 30 min each with 0.1 M phosphate buffer plus 0.1% Na-azide in preparation for mounting. Tissue was mounted in ~1 g of melamine resin consisting of 0.6 g 2,4,6-Tris[bis(methoxymethyl)amino]-1,3,5-triazine (melamine, TGI America, #T2059), 80 mg citric acid monohydrate (Sigma-Aldrich, #C1909), 20 mg of 8,000 MW polyethylene glycol (2%, w/w, Sigma-Aldrich, #89510), 5 mg caffeic acid (Sigma-Aldrich, C0625), 5 mg propyl gallate (Sigma-Aldrich, #02370), and 0.3 ml distilled water. The mixture was liquified by vortexing and then incubating on a horizontal shaker (200 rpm) in an oven at 55^o^C for 1 hr. For mounting, the tissue was rapidly washed 2x with distilled water that was then completely removed. The tissue was bathed in melamine resin for 1 hr at room temperature and then removed and transferred with a fine spatula to a glass slide. 15 µl of free resin was added to the tissue followed by coverslipping (Zeiss, High Performance, 18 x 18 mm, #1½). The resin was cured at 55 ^o^C for 2 days after which it had a refractive index of 1.52.

Primary antibodies, sources, and dilutions are listed below. Secondary antibodies (JacksonImmuno) raised in donkey against mouse (#715-005-151), rabbit (#711-005-152), goat (#703-005-155), guinea pig (#706-005-148), and chicken (#705-005-147) were reacted with NHS-ester dyes using standard procedures (http://abberior-instruments.com/wp-content/uploads/0236_20120316-labeling_protocol.pdf). NHS-ester dyes were ATTO 532 (Atto-tec, #AD 532-31), Abberior STAR 580 (Abberior GmbH, ST580-0002), Abberior STAR 635 P (ST635P-0002), and CF680R (Biotium). Degree of labeling and final antibody concentration were determined with a spectrophotometer using published values for maximal absorption wavelengths, extinction coefficients, and correction factors at 280 nm. Secondary antibody dilution was 1:200. Primary antibodies, species, sources, and dilutions include: guinea pig anti-PSD95 PDZ domain, SySy 124014, (dil. 1:1000); rabbit anti-SLC1A7/EAAT5, Sigma, HPA049124, (1:100); goat anti-CtBP2 (C-16), Santa Cruz Biotechnology, sc-5967 (1:100); rabbit anti-GluR4, EMD Millipore, AB1508 (1:200); mouse anti-GluR5 (E-12), Santa Cruz Biotechnology, sc-393420 (1:500); goat anti-ChAT, Chemicon, #AB144P (1:100); chicken anti-GFP, Abcam, ab13970, (1:1000); chicken anti-bassoon, SySy 141016 (1:500); and rabbit anti-Alexa Fluor 405/Cascade Blue, A-5760 (1:500).

Intravitreal injection used a capsid modified AAV2 containing a single stranded DNA which coded for green fluorescent protein (GFP) under the control of a chicken β-actin promoter^6^. Anesthesia was produced by injecting animals with ketamine (100 mg/kg) and xylazine (10 mg/kg) IP. The target eye received one drop of dilute betadine solution which was immediately followed by one drop of proparacaine (1%). A 27-gauge syringe needle was used to make a hole at a location 1–2 mm below the limbal margin near the medial epicanthus. Virus (10–20 µl; titer equal to 10^12^–10^13^/ml) was injected by inserting a blunt 30-gauge syringe-mounted needle through the access hole into the vitreal chamber. After injection, antibiotic drops were then applied to the treated eye, and the animal was injected with meloxicam (1.0 mg/kg, SC), yohimbine (0.5–1.0 mg/kg, IM), and 0.9% NaCl solution (5–10 ml, SC). The typical incubation period was 3 weeks after which the retinas were removed and processed using glyoxal fixation.

### STED microscopy image acquisition and analysis

Super resolution microscopy was performed on a Leica SP8 3D-STED system using a 100X NA 1.4 oil objective, white light laser for excitation, a 775 nm depletion laser, and z vortex set for isotropic voxel generation under control of LASX software. 3D capture used 20 nm XY and 50 nm Z steps using 16 kHz scan rate, 8 or 16 line average and varied frame accumulation to build up the signal. Image data were denoised using FIJI CSBDeep noise2void trained on each day’s data (https://imagej.net/plugins/n2v). Denoised images were deconvolved in Imaris 9.8.0 using the ClearView module. Deconvolution parameters were: Robust (iterative), 2.0 pre-sharpening gain, 10 iterations, denoising filter equal to 0.7. Specimen and medium refractive index were both set to 1.52. Unless otherwise noted, images are maximum intensity projections.

The analysis procedure used in Fig. 3f had four steps (Supplementary Fig. 6). In step 1, square regions centered around each terminal’s receptor labeling were excised from a binarized (Fiji, default settings) STED image. The size of the square region was 4 μm × 4 μm. Excised images were superimposed and averaged to obtain the spatial profile of GluK1 or labeled BC dendrites beneath the terminal. Averages were converted to 8 bit. Step 2 involved feature extraction by Fourier filtering. A two-dimensional Fourier transform was applied to the average profile, the frequency components with low power were removed, and then an inverse transform was performed. The power cutoff threshold was determined manually for each image and is indicated in the figure panels. In step 3, contour borders were abstracted from the filtered profiles using a Canny edge detection algorithm and a Hough transform, the latter by assuming a circular or elliptical profile. Canny edge detection and Hough transforms were executed using Python OpenCV modules. The fourth step was to quantify the spatial bias of GluK1 labeling or dendrite distribution using moment analysis. Within the region formed by central and peripheral contours, the centroid coordinates were calculated twice, first for a uniform distribution and then for the actual intensity distribution. In both cases, the centroid coordinates (*Cx*, *Cy*) were given by the following equation:

2 $$\left({C}_{x},\, {C}_{y}\right)=\left(\frac{{\Sigma }_{i}{x}_{i}R\left({x}_{i},\, {y}_{i}\right)}{{\Sigma }_{i}R\left({x}_{i},\, {y}_{i}\right)},\, \frac{{\Sigma }_{i}{y}_{i}R\left({x}_{i},\, {y}_{i}\right)}{{\Sigma }_{i}R\left({x}_{i},\, {y}_{i}\right)}\right)$$

where *x*_*i*_, *y*_*i*_ are the *i*-th pixel coordinates of the formed region, and *R*(*x*, *y*) is the pixel value (e.g., the overlap fraction of GluK1). The difference vector between the coordinates obtained from the uniform profile and actual data gives the magnitude and orientation of the bias of the GluK1 or dendritic process distribution.

### Receptor modeling

The glutamate receptor models for cb1a and cb2 were based on a Markov scheme^82^. The behavior of the receptors was represented by transitions between nine discrete states (Supplementary Fig. 9a). The transition rates were different depending on the cone BC type (Supplementary Table 3). Previously published rates were used for the cb2 receptor model^18^. The rate constants for the cb1a cell receptor model are new and were obtained using Particle Swarm Optimization (PSO)^83^. The model output was optimized against receptor responses and parameters during various test glutamate applications. These parameters include individual response rise and decay, EC_50_, IC_50_, recovery τ’s from desensitization, and the percent open probability during long exposures to glutamate (Fig. 5b-d; Supplementary Fig. 8) using a weighted sum of the absolute errors of the real and model data. The optimization process searched for the rate parameter set that minimized the weighting function. PSO was performed by using PySwarms version 1.2.0. Maximal channel open probability was estimated using non-stationary fluctuation analysis from repeated responses of cb1a receptors to an 18 mM glutamate step administered by rapid perfusion. Timing jitter between separate responses in the 20-40 µs range was removed before processing by aligning the traces according to their rising phase.

### MCell synaptic modeling

We use MCell 3.4 and CellBlender 1.0.1 to construct a model synaptic cleft (Supplementary Fig. 9d)^84,85^. The synaptic cleft consisted of 20 μm × 20 μm planes corresponding to the pre- and post-synaptic membranes. The planes were separated by a 16 nm cleft^37,86^. Glutamate molecules were released from a point in the center of the presynaptic plane with a diffusion coefficient of 400 μm^2^/s^18^. There is substantial uncertainty about the number of glutamate molecules in a vesicle in the range of 3000–8000^87^. Therefore, instead of releasing glutamate molecules in increments of “quanta”, we varied transmitter release in increments of 1000 molecules. The presynaptic plane was impermeable to glutamate while the postsynaptic plane was 10% permeable so that lateral glutamate diffusion in the cleft is consistent with previous work^18^. Permeability across the postsynaptic plane is consistent with it being composed of dozens of individual dendritic contacts each separated by an extracellular space. The planar distances of transporters and receptors from the release site was increased to simulate the effect of transmitter flow into the plane through an invagination-like cylinder 200 nm deep by 50 nm wide. Adding a cylinder instead made little difference in the outcome of the simulation. An annular patch (0.02 μm^2^) with 100 cb2 receptors surrounded the release site at a radius of 100 nm, and a square patch (0.04 μm^2^) with 350 cb1a receptors was centered 600 nm away from the release site on ‘basal’ membrane. In addition, a fan-shaped patch with 3200 EAAT5 transporters was placed between those two receptor patches in a region of the model surface corresponding to basal membrane. The transporter model was based on a simple transition between glutamate bound and unbound states in the range bounded by the measured EC_50_ for expressed EAAT5^49^ and the results obtained by rapid perfusion (Supplementary Fig. 2e, f). The forward and backward rates were 10^7 ^M^-1^s^–1^ and 10 or 200 s^–1^, respectively. In some simulations, the transporter patch was moved to the side of the invagination opposite to the receptor patch and in others it was moved in the same direction but beyond the receptor patch.

### Electron microscopy

EM reagents were purchased from Electron Microscopy Sciences (EMS; Hatfield, PA, USA). Animals were euthanized as described above. Once breathing stopped, trans-cardiac prefusion of the following physiological saline began (in mM): NaCl 120, KCl 3, MgSO_4_ 2.5, glucose 10 CaCl_2_ 2, NaHCO_3_ 25, equilibrated with CO_2_/95% O_2_ to a pH of 7.4, and administered at a flow rate of ~15 mL/min. Next, trans-cardiac infusion of the following aldehyde solution: 0.6% paraformaldehyde and 2% glutaraldehyde in 1X PBS, was delivered via trans-cardiac infusion for 10 min. The first 10 ml of the fixative was perfused in the first 1 min, and the remaining 10 ml were delivered over 5 min while the superior vena cava was clamped to increase perfusion pressure in the head. The eyes were removed, and the superior and inferior retina was trimmed away from the peripheral retina. Retinal pieces were immersed in the aldehyde fixative solution and incubated on ice overnight. After 24 h, retinal pieces were washed in 8% sucrose in 1X PBS at 4 °C to remove free aldehydes. The retinal pieces were then equilibrated in 2X PBS for 20 min and brought to RT. The tissue was postfixed with 2% OsO_4_ in 1X PBS for 60 min at RT on shaker table and washed in 1X PBS and H_2_O. En bloc counterstaining and dehydration were performed at RT and carried out as previously described^44^ with a few minor changes. Counterstaining consisted of three successive 15 min exchanges in 40 mM maleate buffer (pH 5.2) followed by a 30 min incubation in 2% uranyl acetate (w/v in maleate buffer) in the dark. Next, the tissue was rinsed in pure H_2_O immediately before starting the following dehydration series in EtOH: 50% EtOH 10 min, 70% for 10 min, 90% for 15 min, and 100% 10 min. Finally, the tissue was dehydrated in 100% propylene oxide (PPO) for 10 min. Infiltration into EPON812 was carried out by placing samples on a tissue rotor followed by a series of infiltration steps: (1) 1:1 PPO:EPON812, 3 hrs RT; (2) 70% EPON812 in PPO for over 5 hrs at RT; (3) 100% EPON812 for 3 h RT. Infiltrated retina was further dissected such that it could be placed flat-mounted in molds filled with freshly prepared 100% EPON812. Samples were placed in a 40 °C oven and cured overnight. The temperature was then increased to 65 °C for at least 12 h. Only portions of superior retina were further sectioned and analyzed, which is the same region of the retina that we recorded from with electrophysiological physiological methods. Ultrathin sections were made as described previously and counter stained in lead citrate^44^. Sections were made vertically and tangentially through the retina: perpendicular to the OPL, and tangential to the OPL. Transmission electron microscopy was performed with an FEI Tecnai Spirit G2 (Center for Advance Microscopy, Northwestern University School of Medicine). Analysis of EM images was performed using ImageJ software (NIH, Bethesda, MD, USA).

### Statistics and reproducibility

Additional statistics for Fig. 10b.

Above, for cb1b and cb2 current responses (the average of 3 complete stimulus sequence repeats) during all decrements, the hypothesis that the levels are the same was rejected (cb1b: critical value = 1.96, right to left t value = 31.27, 23.52, 53.19, 82.02, 111.63; cb2 t value = 23.55, 21.38, 57.74, 86.27, 124.88; *n* = 2000 points (i.e., 400 ms) before versus 2000 during the stimulus; two-tailed unpaired *t* test). Cb1b and cb2 normalized current responses were different from each other (critical value = 1.65, right to left t value = 3.00, 10.66, 35.97, 31.87, 67,38; two-tailed unpaired *t* test).

Upper middle, Levene’s Mean Statistic (LMS) tests the hypothesis, H0, that the variance before and during a light decrement are equal (α = 0.05, critical value = 3.844; cb1b, right to left, LMS = 6.54 H0 rejected but Δvar was -0.12 pA^2^, 3.484 H0 accepted, 53.186, 590.245, 1067.06; cb2 LMS = 115.14, 306.51, 407.96, 988.35, 978.58).

Lower middle, unitary response amplitude was calculated separately for each of the three stimulus sequence repeats and plotted at each step-to intensity. Mean±S.E. was also plotted. A two-tailed one sample t test was used to determine whether the size of the cb1b cell unit was different from 0 (cb1b: right to left, p=not calc., 0.8922, 0.0738, 0.1025, 0.0005). When the 6 unitary response amplitudes during decrements 4 and 5 were grouped together, the mean was different from 0 (*p* = 0.0017). During the smallest decrement, the mean cb2 cell unit was not significantly different either from 0 (*p* = 0.1071) or from the mean value of the other 4 decrement responses (7.9 pA, p = 0.4849; two-tailed one sample *t* test).

Below, mean cb1a/b cell unit amplitude different from decrement 5 (right to left, *p* = 0.0178, 0.0045, 0.0052, 0.3048; two-tailed unpaired *t* test).

### Reporting summary

Further information on research design is available in the Nature Portfolio Reporting Summary linked to this article.

### Supplementary information

Supplementary Information

Reporting Summary

Description of Additional Supplementary Files

Supplementary Movie 1

### Source data

Source Data

## Data Availability

Source data are provided with this paper for all electrophysiological traces and graphs appearing in figures. The electrophysiological traces provide a minimal basis for interpreting and verifying key results. Large image stacks (TIF) surface mesh files (STL) and associated data files are available at the Dryad repository (doi:10.5061/dryad.0p2ngf25g). Additional raw data will be shared by the corresponding author upon request. Source data are provided with this paper.

## Code availability

The code used to model signaling at the cone synapse is published at Zenodo and is accessible via the Dryad link at the landing page under “Related works”.
