# Author Correction: Mechanisms of simultaneous linear and nonlinear computations at the mammalian cone photoreceptor synapse

**DOI:** 10.1038/s41467-024-45588-2

**Published:** 2024-03-20

**Authors:** Chad P. Grabner, Daiki Futagi, Jun Shi, Vytas Bindokas, Katsunori Kitano, Eric A. Schwartz, Steven H. DeVries

**Affiliations:** 1 Institute for Auditory Neuroscience, University Medical Center Göttingen, 37075 Göttingen, Germany; 2 Synaptic Nanophysiology Group, Max Planck Institute for Multidisciplinary Sciences, 37077 Göttingen, Germany; 3 College of Information Science and Engineering, Ritsumeikan University, Shiga, Japan; 4 Center for Systems Visual Science, Organization of Science and Technology, Ritsumeikan University, Shiga, Japan; 5 Ritsumeikan Global Innovation Research Organisation, Ritsumeikan University, Shiga, Japan; 6 Department of Ophthalmology, Northwestern University Feinberg School of Medicine, Chicago, IL 60611 USA; 7 Dept of Pharmacological and Physiological Sciences, The University of Chicago, Chicago, IL 60637 USA

**Keywords:** Retina, Neurotransmitters

Correction to: *Nature Communications* 10.1038/s41467-023-38943-2, published online 16 June 2023

The original version of this Article omitted “DFG CRC1286 for Quantitative Synaptology at the University of Göttingen (CPG)” in the Acknowledgments. The corrected Acknowledgments reads:

“This work was supported NIH R01 EY012141, an unrestricted grant to the Dept of Ophthalmology from Research to Prevent Blindness, an International Travel Award from RPB (S.H.D.), JSPS KAKENHI grant #19H01140 (K.K. and D.F.), and DFG CRC1286 for Quantitative Synaptology at the University of Göttingen (CPG). Imaging and data analysis was performed at the University of Chicago Integrated Light Microscopy Core RRID: SCR_019197. We wish to thank Raina DeVries (University of Chicago) for 3D surfacing of super-resolution images, Peter Sterling (University of Pennsylvania) for helpful comments on the manuscript, and Yongling Zhu (Northwestern University) for AAV manufacture.”

This has been corrected in the PDF and HTML version of the article.

The original version of this Article also omitted an affiliation for the author Chad P. Grabner, “Collaborative Research Center 1286, University of Göttingen, Göttingen, Germany”.

