# Supplementary material for: Mechanisms of simultaneous linear and nonlinear computations at the mammalian cone photoreceptor synapse
Source: Nat Commun. 2023 Jun 16;14:3486. doi: 10.1038/s41467-023-38943-2 (PMC10276006; doi:10.1038/s41467-023-38943-2)
Supplement: Supplementary file 1 — Supplementary Information [file 41467_2023_38943_MOESM1_ESM.pdf]

Supplementary information

Supplementary Figures 1-10

Supplementary Tables 1-3

Mechanisms of simultaneous linear and nonlinear computations at the mammalian cone photoreceptor synapse

Chad P. Grabner, Daiki Futagi, Jun Shi, Vytas Bindokas, Katsunori Kitano, Eric A. Schwartz, Steven H. DeVries

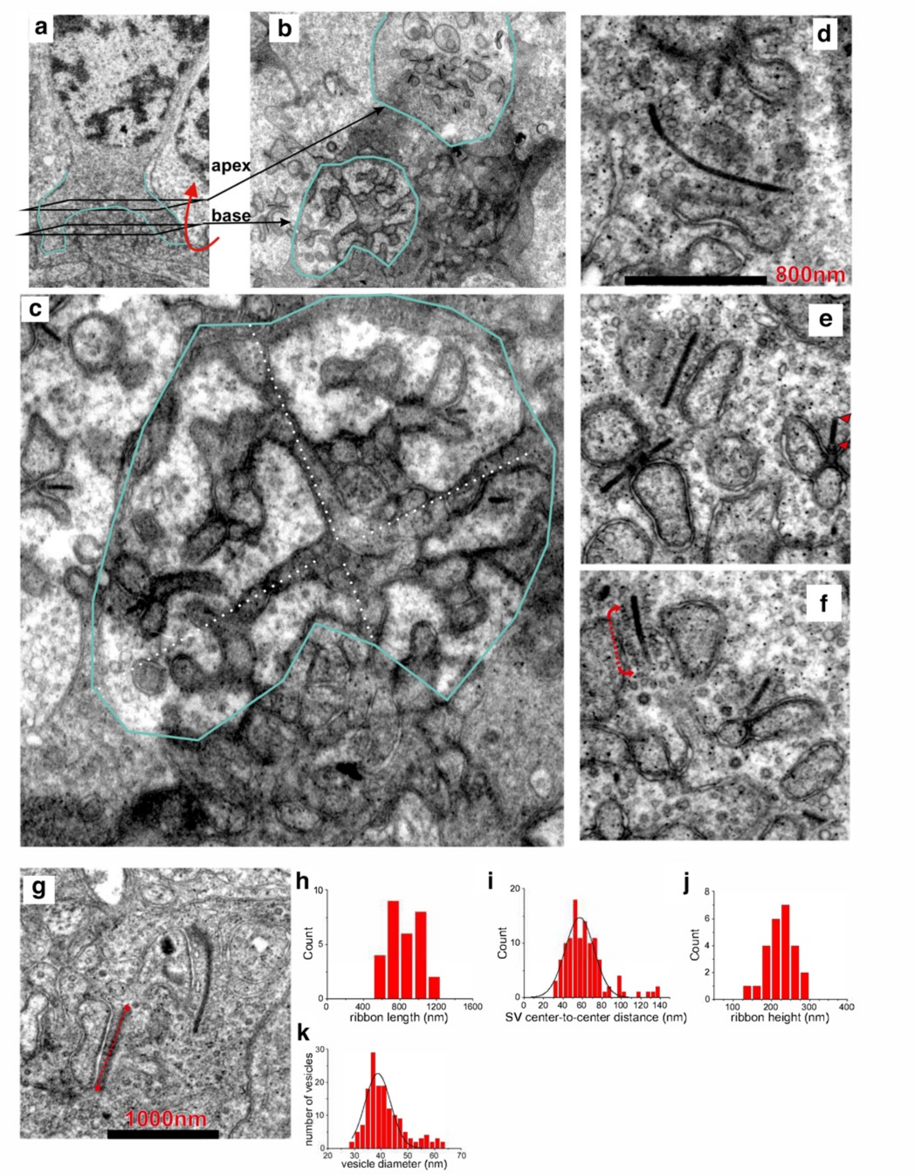

Supplementary Fig. 1. Analysis of vesicle density on ribbons. **a.** Electron micrograph showing a vertically oriented cone and terminal. The terminal base has a concave profile (cyan line). **b.** Tangential section through the cone terminal region showing two profiles. The upper terminal profile is sectioned through the apex of the concavity and shows a cluster of ribbons. **c.** Lower terminal profile in *b* at higher magnification. The section captures the cone near the base of the pedicle. The central region is dominated by postsynaptic dendrites. The dashed lines highlight the major dendritic branches. **d.** An image used to estimate ribbon length. **e., f.** Example micrographs used to determine ribbon height and vesicle density on the ribbon (*d*-*f*, are presented at the same scale). Arrows in *e* denote a ribbon that was sectioned vertically relative to the arciform density and synaptic triad. Arrows and dashed line in *f* denote a ribbon with vesicles aligned along one side. **g.** Two ribbons, captured in a horizontal section, used to estimate ribbon length. **h-j.** Histograms of ribbon length (mean = 824 ± 30 nm; n = 29 ribbons), the distances between synaptic vesicles on the ribbon (57.0 ± 0.9 nm; n = 116 vesicles), and ribbon height (225 ± 7 nm; n = 25 ribbons). **k.** Histogram of vesicle diameters (consistent with a single vesicle C_m of 43 attofarads[1]).

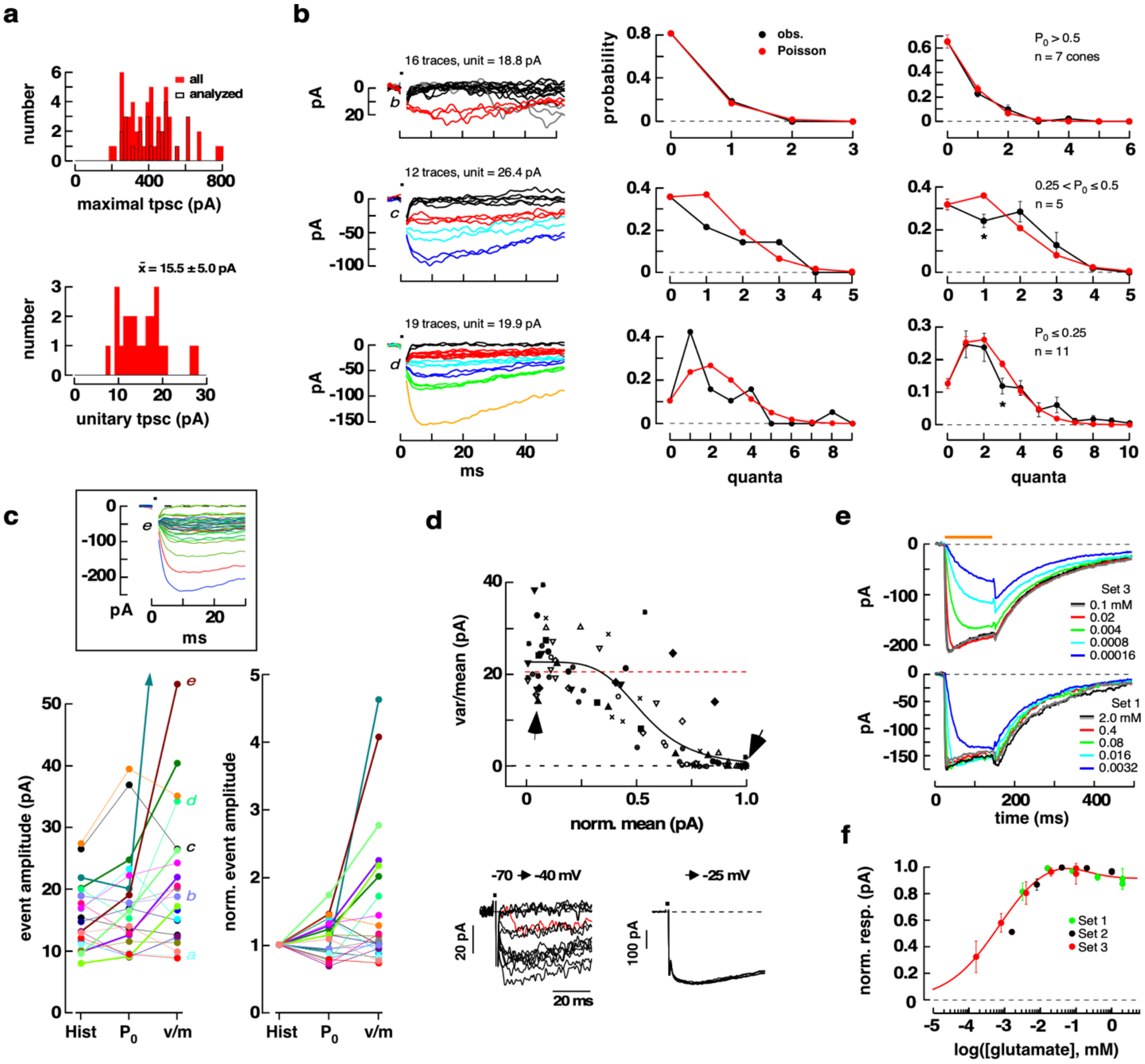

Supplementary Fig. 2. Properties of the cone tpsc response. **a.** Histograms of the maximal cone tpsc response to a 1 ms depolarization in the set of 55 cones (above) and the unitary response amplitudes (below) obtained from a subset of 24 cones (black open bars) by fitting their amplitude histograms (15.5 ± 5.0 pA, mean ± S.D.). The subset of 24 cones (peak tpsc amplitude = 420.1 ± 113.3 pA) was selected from the set of 55 cones (tpsc amplitude = 409.0 ± 137.1 pA) by applying the following exclusionary criteria: 1) no response failures in the sequence (13 cones); 2) a high spontaneous event rate that caused evoked and spontaneous events to frequently overlap (10 cones); and 3) low maximal peak amplitude (<200 pA; 2 cones). For six additional cones, the amplitude histogram fits were indeterminate. **b.** Tpsc amplitudes were Poisson distributed especially during steps to relatively hyperpolarized voltages that had a high probability of response failure, P_0. Predicted distributions were calculated from the measured P_0 ranging from high (top row), to medium (middle), to low (bottom). Left column: consecutive tpsc responses during a train of steps to the same voltage color-coded according to amplitude histogram-defined quantal levels. Responses from 3 biologically independent cones. Center column: observed and predicted quantal content probabilities for the responses in the left column. Right column: aggregate data. Responses were grouped and averaged according to ranges of P_0. Asterisks denote values that were different at the p=0.0052 and p=0.0155 levels (top and middle, respectively; two-tailed unpaired t test). In the middle and lower panels, a slight but not statistically

significant skewing of observed responses to higher levels may be due to multiquantal release. Data is present as mean ± S.E. **c.** Category plots comparing the estimates of unitary event amplitude obtained by three different methods: amplitude histogram, P_0, and variance to mean (v/m) ratio. Colored lines connect results from the same cone. Letters denote the results from analyzing the trains plotted in *b* (labeled b-d), Fig. 2 (labeled a), and the *inset* (labeled e). Points were plotted without (left) and with (right) normalization to the values obtained from the amplitude histogram. To estimate an effective unitary event size, we relied on amplitude histograms and measurements of P_0 rather than the response variance to mean ratio, which can be distorted by the presence of multiquantal events. **d.** Plot of tpsc variance to mean ratio versus normalized tpsc amplitude for 13 cones (data from each cone is denoted by a different symbol; normalization was against the peak tpsc amplitude for the cone; the peak tpsc for the 13 cones was 465.0 ± 29.0 pA; mean ± S.E.). A sigmoid fit to the aggregate data had a maximum value of 22.7 pA and was constrained to approach zero at high mean values. The sigmoid fit had a ½ decay point at 54% of the mean response. The curve crossed the 90% level (red dashed line) at 34.2% of the maximal response. This percentage corresponds to a current of ~160 pA relative to the mean peak tpsc value of 465 pA, which is equivalent to ~10 average (*i.e.*, 15.5 pA) quantal units. Arrows denote the points (upright filled triangles) associated with the sets of traces that show the responses of a single cone during minimal (*inset*, bottom left) and saturating (*inset*, bottom right) 1 ms pulses. The red trace shows a spontaneous tpsc that immediately followed a failure. The 13 cones in the plot were selected from the 24 in the sample based on having sufficient data points at intermediate current levels to provide information on the sigmoidal decay. **e.** Glutamate concentration dependence of the transporter anion current measured in excised cones by rapid perfusion. Panels show responses during rapid steps from control to solutions with different concentrations of glutamate (orange bar). Glutamate concentrations ranged from 0.1 to 1.6×10^{-4} mM (top panel, concentration set 3) to 2.0 to 3.2×10^{-3} mM (bottom panel, set 1). **f.** Peak response versus glutamate concentration plotted for the three concentration ranges (mean ± S.D.). Data was from 3, 3, and 4 cones in sets 1, 2, and 3, respectively. Normalization was to the largest peak current value in each experiment. The curve is the sum of two Hill equations, one positive-going ($x_{½}$ = 0.71 μM, n=0.6, where x is glutamate concentration) and the other negative-going ($x_{½}$ = 0.15 mM, and n=1 by constraint). The transporter current response saturated at glutamate concentrations >20 μM. The concentration range of the saturating response is inconsistent with the presence of a low affinity transporter current[2]. In a separate set of rapid perfusion experiments, cones were switched from control to either 5 μM glutamate or 5 μM glutamate plus 100 μM dihydrokainate (DHK). DHK produced a small (-5.62 ± 6.8%, n=4) but not significant reduction in transporter current (p = 0.1994). Similar concentrations of DHK markedly decrease transporter currents in salamander cones and bipolar cells, which have a sizeable EAAT2-mediated component[3].

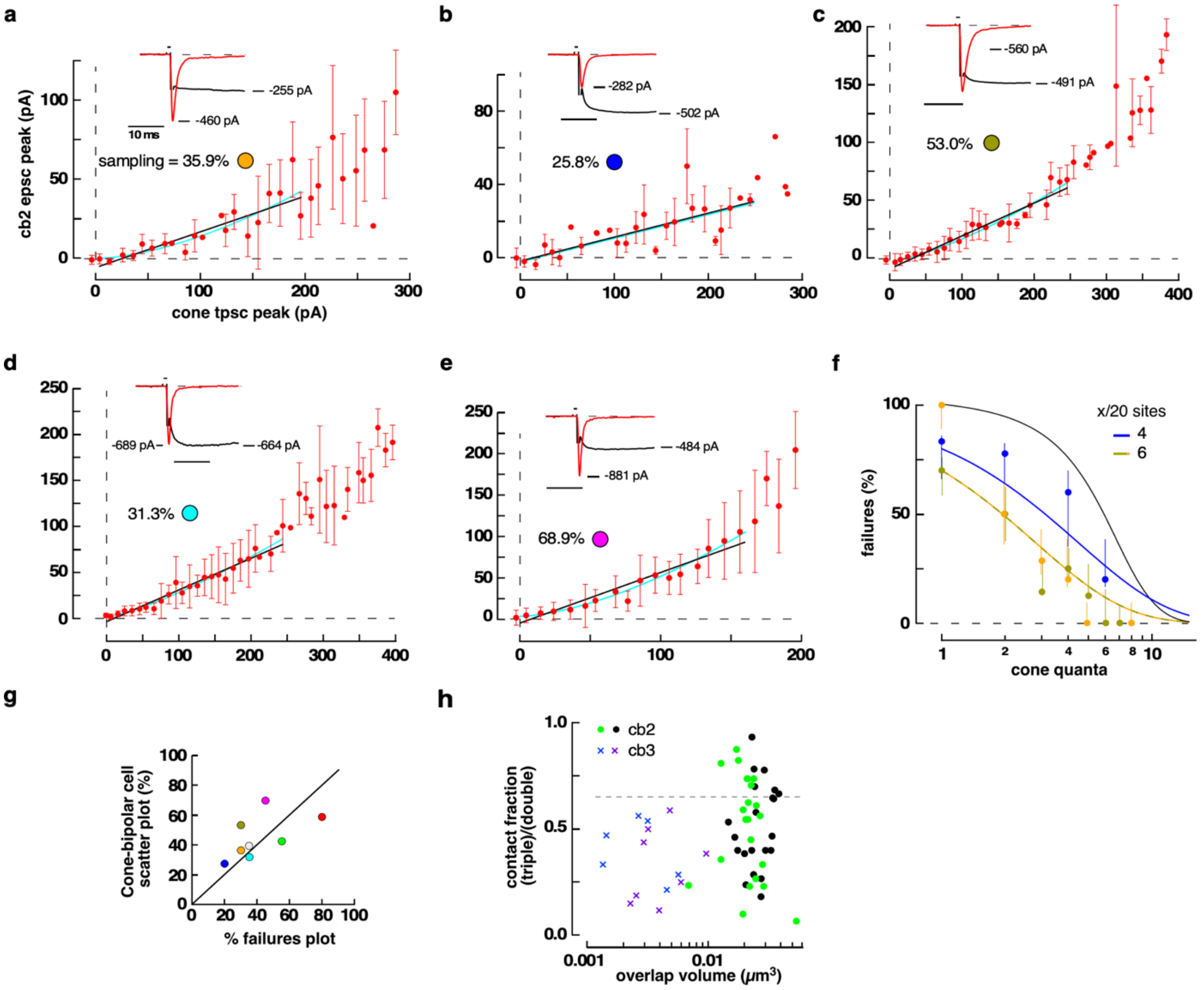

Supplementary Fig. 3. a-e. Scatterplots for 5 additional cone to cb2 cell pairs. Data points are averages over 10 pA bins of cone tpsc amplitude. The mean ± S.D. for the BC epsc peak in each bin is shown. Points that lack error bars resulted from a single measurement. Point location on the x-axis is also an average value relative to the 10 pA bin width. Least squares fit to linear (black) and power-law (cyan) equations are shown over the cone-linear range. Sampling percentage was calculated from the linear slope after normalizing for cone and cb2 BC unit amplitude. **a.** Linear fit preferred, $p = 0.1476$ (F test). **b.** Linear fit preferred, $p = 0.8183$. **c.** Power-law fit slightly preferred, $p = 0.0422$, exponent = 1.35 (F test). **d.** Linear fit preferred, $p = 0.0861$ (F test). **e.** Power-law fit preferred, $p = 0.0002$, b = 1.59 (F test). Insets show the maximal cone tpsc and cb2 cell epsc for each pair. **f.** Percent failures versus cone quantal content plots and fits for three of the pairs which are biologically independent. Confidence intervals (50%) are related to the number of trials and percent failure (see Methods). **g.** Comparison of percent sampling of cone release sites by cb2 cells obtained by using either the slope fit method or the percent failures method. Corresponding cone to cb2 cell pairs are denoted by the large colored circles in Figs. 2f,g and Supplementary Fig. 3a-f. **h.** GFP-labeled cb2 BCs like that shown in Fig. 1k-n were used to count the number of contacts between a cb2 cell and cone terminals that are central in the dendritic field. For each cone in the cb2 dendritic field, the total number of ribbon release sites was estimated from close associations (using a minimal surface to surface distance criterion) between GluA4 and Ribeye and designated as 'doubles'. A subset of release sites was designated as contacted (*i.e.*, 'triples') based on further close association between GluA4 and dendritic labeling. The contact fraction for each cone terminal equaled the number of triples divided by the number of doubles. While some cb3 cell dendrites also qualified as triples based

on surface-to-surface distance, a second criterion, the overlap volume between the dendritic and GluA4 surfaces distinguished between cb2 and cb3 cell contacts. In accordance with BC dendritic tiling[4], we assumed that central cones in the dendritic field were solely innervated by the labeled cb2 cell and thus ordered the cone terminals according to contact fraction and focused on only the top quartile. The top quartile had a contact fraction of 80.7 ± 4.7% (n = 2 cb2 cells with 19.0 ± 4.4 presynaptic cones per cell) and corresponded to central cones. A comparable analysis of labeled cb3 cell contacts (n = 2 cells) showed less volume overlap and a lower contact fraction. Dashed line indicates a contact fraction of 0.65. Different colors and symbols correspond to different BCs.

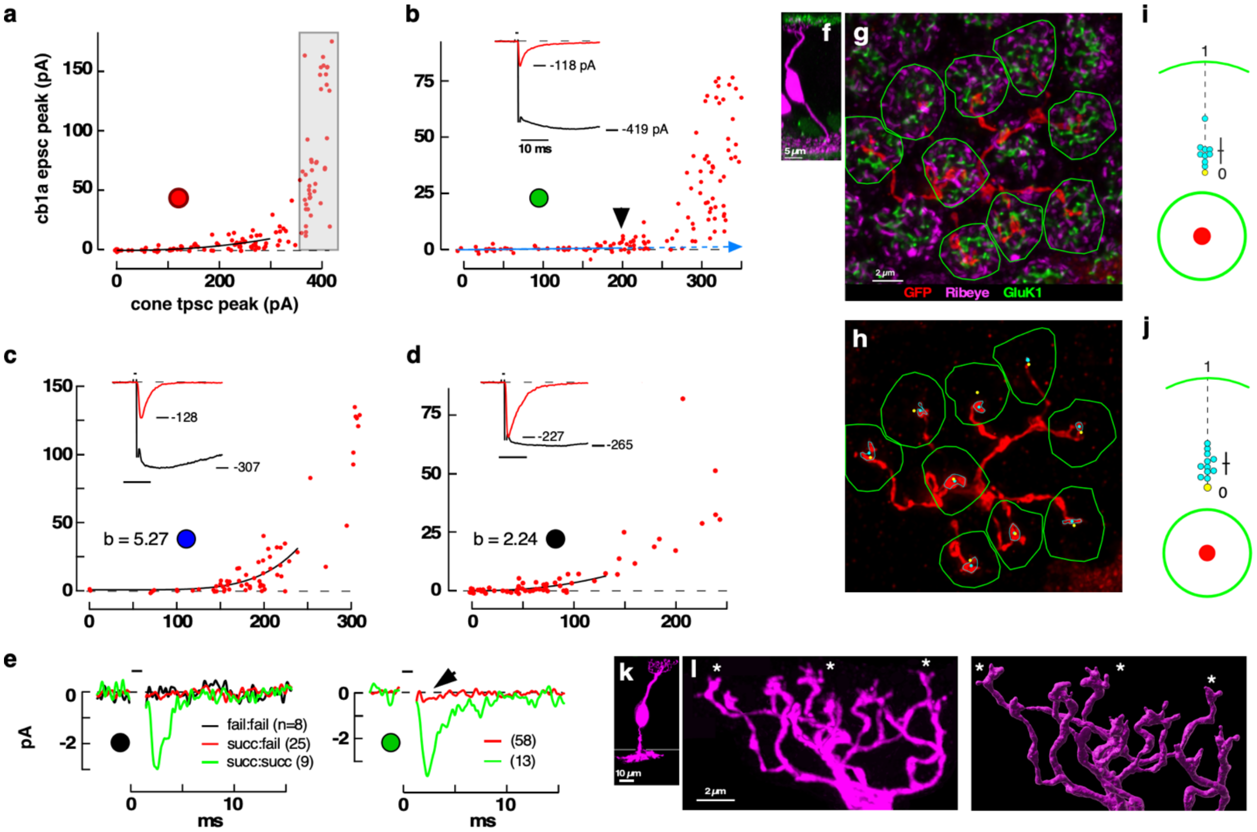

Supplementary Fig. 4. **a.** Full range scatterplot from the cone to cb1a cell pair in main text Fig. 3a,b. The gray rectangle encompasses a region of the plot where the cone transporter saturates. **b-d.** Scatterplots for 3 additional cone to cb1a cell pairs. Power law fits (black curves, where feasible, in the cone-linear range) and exponents are shown. The corresponding data sets in main text Fig. 3g are indicated by the large colored circles. Insets show the maximal cone tpscs and BC epscs. When extrapolated, the blue line in *b* fitted to the points between 0 and 7 quanta (black arrowhead) predicts a peak quantal content of 1801 vesicles. **e.** Analysis of cb1a cell response failures for evidence of 'hidden' epscs. The left panel shows traces from the cb1a cell in *d*. The right panel shows traces from the cb1a cell in *b*. To test for hidden events, traces meeting the following criteria were averaged: BC failures in the presence of a cone failure to release were assumed to represent authentic response failures (black); small responses in the cb1a cell accompanied by a cone event (success:success; green trace) provide an epsc time course template; finally, cb1a cell currents during small cone release events that were designated as failures were averaged to search for consistent small events. Number of traces averaged in each group shown in parenthesis. Deviations from baseline during cone release observed in the right panel (arrow) suggest that some responses classified as failures in this cone to cb1a cell pair may contain small epscs. There were too few events meeting the 'fail:fail' criterion in the pair on the right for an informative average. **f**. Confocal image of cb1a BC (cross-sectional view from a wholemount image stack). **g-h.** STED super-resolution wholemount view of the cell with the borders of the cone terminals, as judged from the Ribeye and GluK1 labeling patterns, delimited with green polygons. Centroids were calculated from the polygons for each contacted cone terminal (yellow dots corresponding to green polygons) and each dendritic terminal (cyan dots corresponding to cyan polygons). **i**. The effective radius of each terminal polygon was calculated from a circle of the same area and used to normalize the distance of the contact centroid from the terminal centroid. The plot in the upper part of the panel shows that the contact centroids for the cb1a cell in *f-h* were displaced from the terminal centers by a normalized distance of 0.175 ± 0.126 (mean ± S.D., different from 0, p = 0.0031; two-tailed one sample t test). The central contacts occupied 3.1 ± 2.1% of the terminal area (lower part of the panel). **j.** A similar analysis

of the cb1a contacts for the cell in Fig. 1e-i also supports a centralized contact location (normalized distance = 0.219 ± 0.104; significantly different from 0, $p = 0.0001$; two-tailed one sample t test) and small area ratio (2.9 ± 2.1%). **k.** Confocal image of different cb1a cell in a retinal slice. **l.** Collapsed STED image stack showing the dendritic tree (left). A surface fit to the dendritic tree (right) shows terminal bulbs with up to 5 thin extensions. Asterisks indicate terminal dendrites shown in main text Fig. 1j.

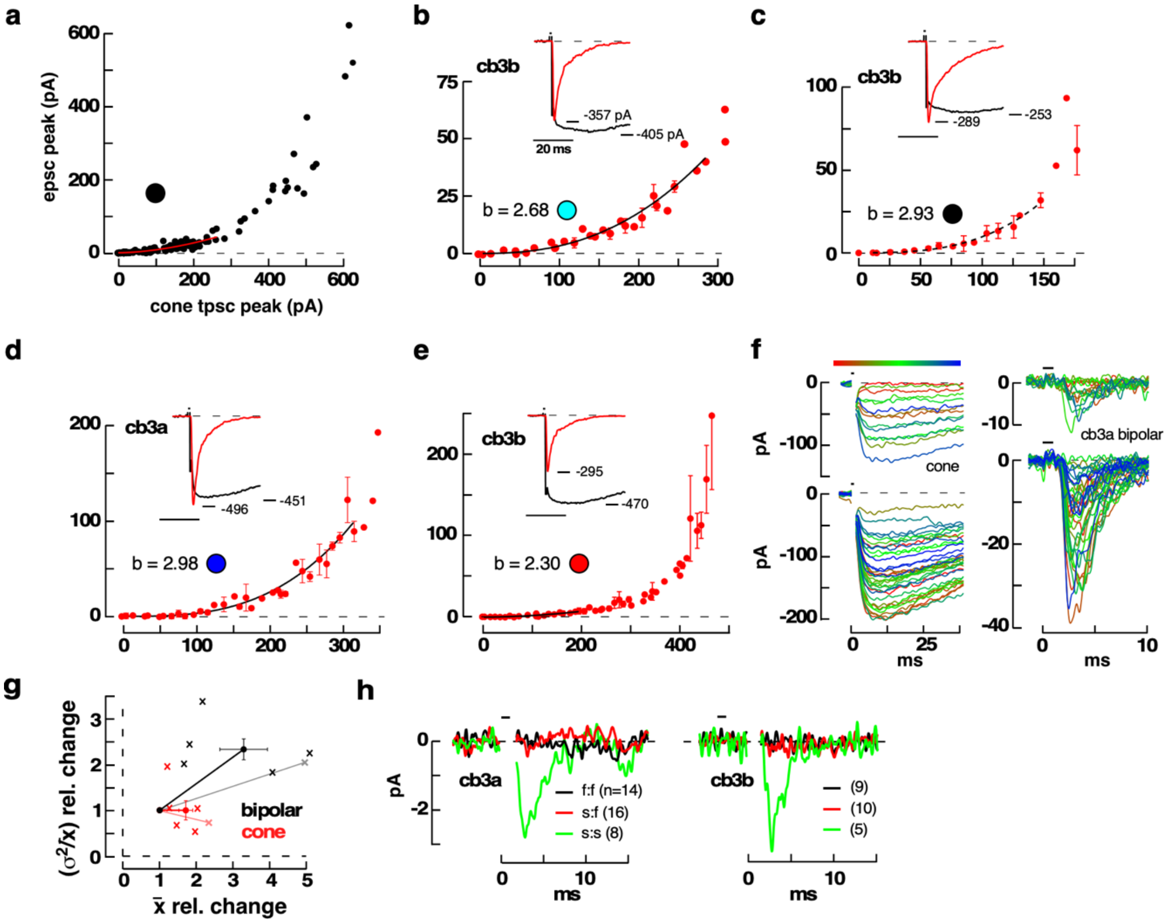

Supplementary Fig. 5. Properties of cb3 cell responses during cone depolarization. **a.** Full range scatterplot from the cone to cb3a cell pair in main text Fig. 3b. **b-e.** Scatterplots for 4 additional cone to cb3 cell pairs. Power-law fits (black curves; significantly better in all four cases $p < 0.0001$), exponents, and corresponding data sets in main text Fig. 3e are indicated. The dashed curve in *c* spans the cone-linear range assuming a standard event size of 15 pA. Insets show the maximal cone tpscs and BC epscs. **f.** Paired responses obtained from a cone and cb3a BC during two stimulus epochs (same cell as in Fig. 3b). A sequence of depolarizing steps to -30 mV elicited cone tpscs that ranged up to -125 pA with a mean of -54.8 pA (upper left). The postsynaptic cb3a cell (upper right) displayed a combination of responses and failures with a mean amplitude of -2.8 pA and an effective unit size of 3.1 pA (here calculated from $\sigma^2/\bar{x}$). The same voltage step during an earlier recording epoch produced a series of cone tpscs with a larger mean amplitude of -133.2 pA (lower left). Associated BC responses (lower right) had a mean amplitude of -33.9 pA and an effective unit size of 17.1 pA. **g.** We tested whether the ~5.5-fold increase in the effective BC unit size, 17.1 versus 3.1 pA, was associated with a comparable increase in effective unit size in the cone response, as might occur if there was an increase in the proportion of multiquantal events. In this test, we used $\sigma^2/\bar{x}$ as a qualitative measure of response amplitude, both for cone tpscs and BC epscs, and calculated the relative change between stimulation epochs in the same recordings. The larger amplitude epoch was selected to have cone responses within the linear summation range (average tpsc amplitudes = 121.8 ± 33.7 pA, n = 6 cone to cb3 cell pairs). Between epochs, mean cone responses increased by a factor of 1.71 ± 0.19 (red points and lines; mean ± S.E.; $p = 0.0135$ two-tailed one sample t-test; x's show individual data points) while mean BC responses increased by a factor of 3.29 ± 0.65 (black points and lines; $p = 0.0169$ two-tailed one sample t-test; ratio difference is significant, $p = 0.0156$, Wilcoxon signed test for paired data). The 1.71-fold increase in the cone response occurred in the absence of a change in the variance to mean ratio (1.0 ± 0.21) suggesting an underlying increase in the quantal content

of the average tpsc without a change in effective unit amplitude. In contrast, the 3.29-fold increase in mean BC epsc amplitude was accompanied by a 2.33 ± 0.23-fold increase in the variance to mean ratio suggesting that an increase in the mean response was associated with both an increase in unit size and quantal content. Gray and pink lines and points correspond to the responses in *f*. **h.** Analysis of two cb3 cell responses categorized as failures for evidence of small epsc responses. Same format as Supplementary Fig. 4e. The red trace averages suggest that categorized response failures in cb3 cells do not consistently contain small epscs.

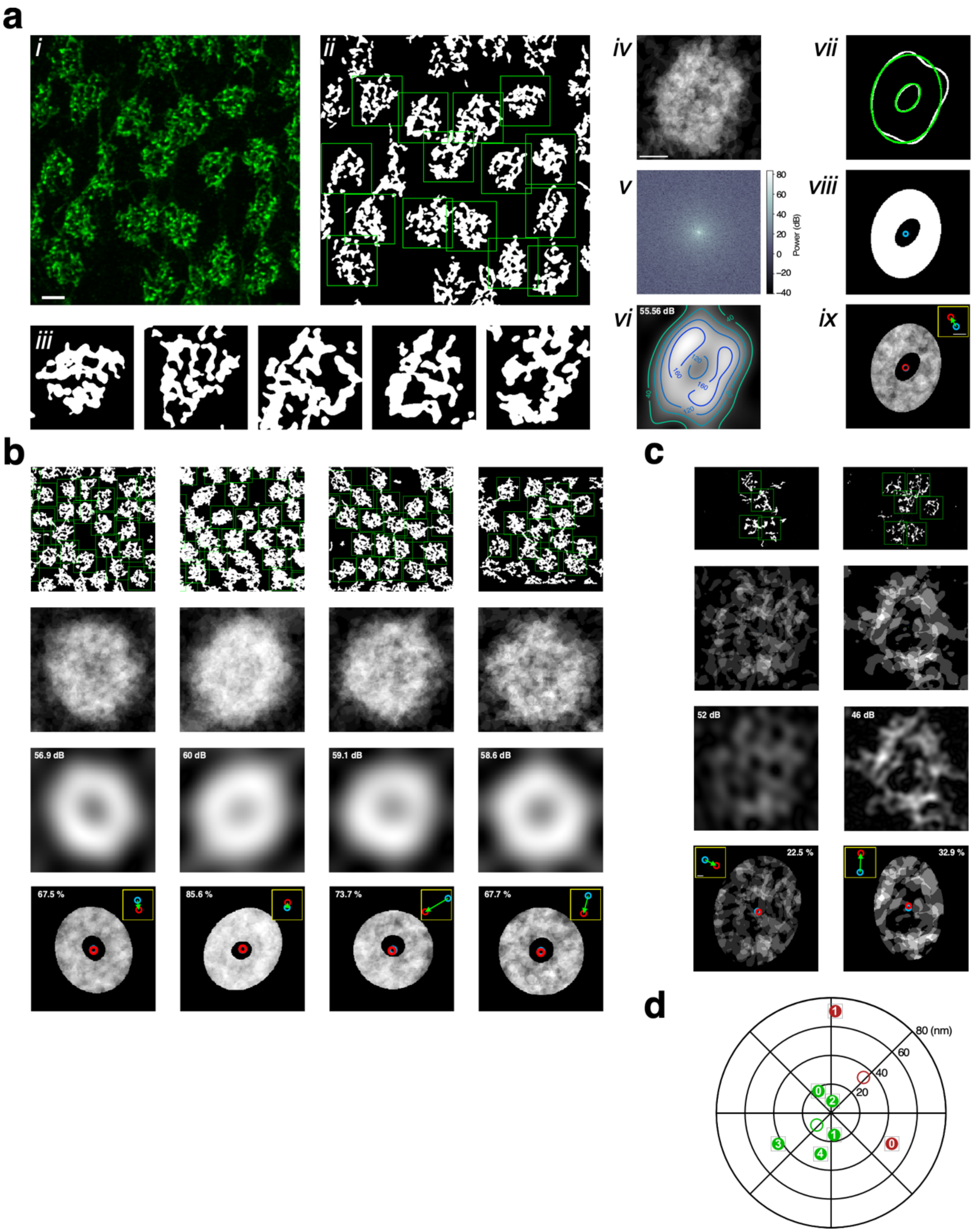

Supplementary Fig. 6. Density of coverage of GluK1-containing receptor and cb3 cell dendrites at the cone terminal. Both cb3a and cb3b cells express KARs containing GluK1 subunits. **a.** (*i*) Maximum intensity projection of GluK1 immunolabeling in a retinal whole mount (scale = 2 µm; larger field of the image shown in Fig. 1o). (*ii*) Binarized image with identical squares centered around selected terminals. (*iii*) Examples of binarized GluK1 labeling at individual terminals. We summed image squares, point by point, without rotation on the assumption that radial differences, if they exist, would have the same relative orientation (*e.g.*, toward the optic nerve head) in a small retinal region. (*iv*) Average of individual squares (same as main text Fig. 3f, upper). (*v*) 2D power spectrum for determining filter cut-off. Components with power less than 55.56 dB were removed. (*vi*). Fourier-filtered image of the average from *iv* showing contour lines. Gray scale is

from 0 to 255. (*vii*) Ellipses were fitted to outer and inner profiles using a Hough transform. Smoothed profiles consisted of a circular or elliptical ring with an average density of 71.1 ± 9.3% (mean ± S.D., n = 5; Supplementary Table 1) and a central dimple with a density of 63.0 ± 13.2%. (*viii*) Location of the unweighted centroid (green circle; see Methods, Eq. 2). (*ix*) Location of the weighted centroid (red) and the displacement vector (green arrow). A weighted vector sum of the density within the ring had X-Y coordinates very close to the unweighted mean (Supplementary Table 2) consistent with a uniform radial density. Scale bar for the inset is 20 nm. **b.** Binarized GluK1 labeling from 4 additional retinas (1st row). Average profiles (2nd row); filtered profiles with the filter cut-offs appended (3rd row); elliptical profiles; and centroid displacements (4th row). Percentages refer to the average gray scale intensity within the ring. **c.** Selected cb3 cell dendritic terminals beneath a cone, excised, averaged, and analyzed as in *b*. Coverage is ring-like and symmetric. **d.** Polar plot of weighted centroid displacement obtained from five images with GluK1 labeling (filled green circles, the open circle is the average). Centroid displacement locations for two of the images that contained cb3 cells (red circles) showing a relatively even distribution under the terminal. Since the dendrites of BCs within a type tile the retina with minimal overlap[4], each cone should receive innervation from the equivalent of one cb3a and one cb3b cell. This dual innervation accounts for nearly all GluK1 labeling insofar as cb1 cells make relatively few cone contacts and have weak GluK1 labeling. The high density of GluK1 labeling beneath a cone terminal combined with the ring-like coverage of cb3 cell dendrites supports the idea of comprehensive sampling of cone transmitter release sites.

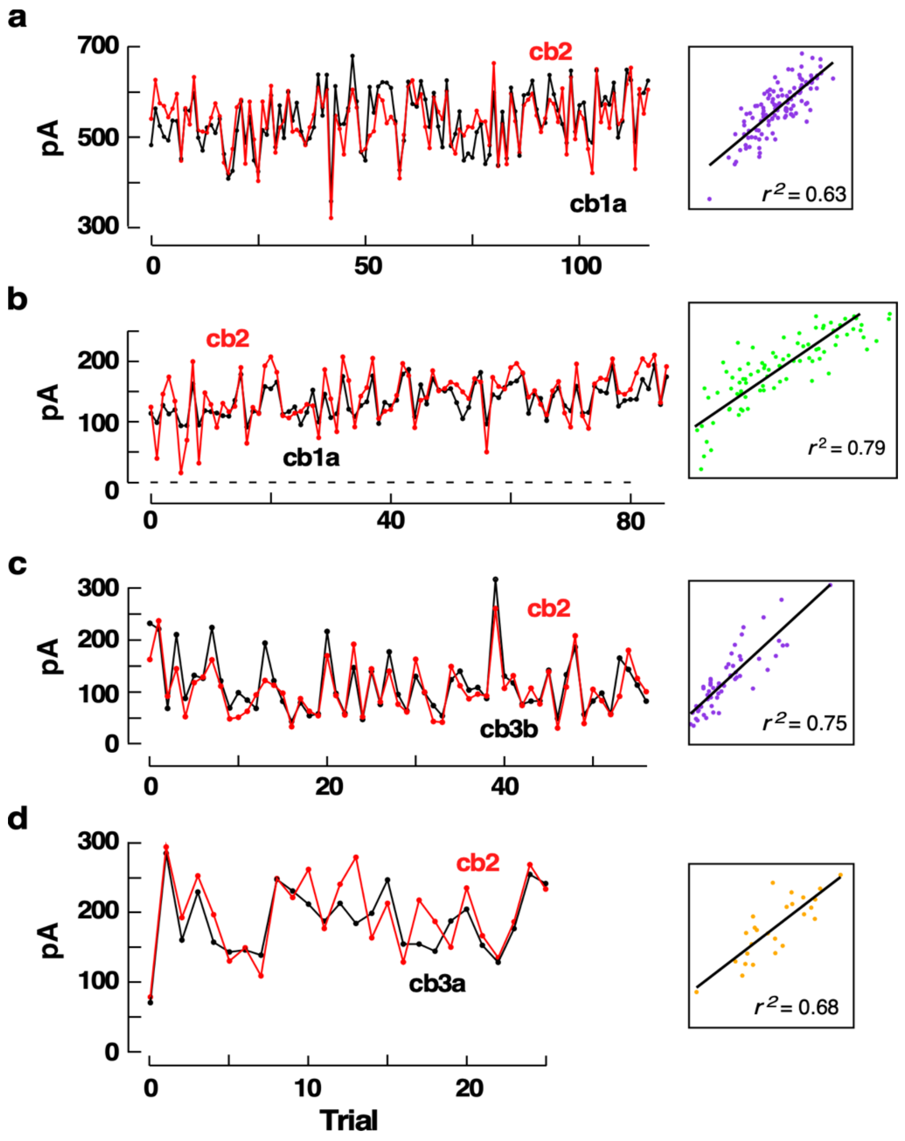

Supplementary Fig. 7. Correlated responses in postsynaptic BCs during depolarization of a common presynaptic cone. **a**. Trial to trial variations in the simultaneous responses of a cb1a and cb2 cell (data from the pair in main text Fig. 4a, left) during a single epoch of cone depolarization. The epsc peak values of the cb1a cell were scaled by the slope and y-intercept according to the equation y = 190 + 5.7x and superimposed on the values for the cb2 cell. *Inset.* The responses had a correlation coefficient, r^2, of 0.63 (point color corresponds to Fig. 4a, left; r^2 is scaling independent). **b.** Data from the cb1a and cb2 cell pair in Fig. 4a (right). The peak epsc responses in the cb1a cell were scaled by y = 90 + 0.8x. **c.** Data from the cb3b and cb2 cell pair in Fig. 4b (left). The cb3b cell peak responses were scaled by y = 40 + 1.2x. **d.** Data from the cb3a and cb2 cell pair in Fig. 4b (right). The cb3a cell peak responses were scaled by y = 31 + 2.6x. Assuming that the release at neighboring invaginations is uncorrelated, simultaneous responses in the postsynaptic BCs should be uncorrelated if sampling is exclusively from different ribbons but completely correlated if sampling is from the same set of ribbons. The range of r^2 values from 0.63 to 0.79 is consistent with overlap in sampling of release sites in a cone terminal.

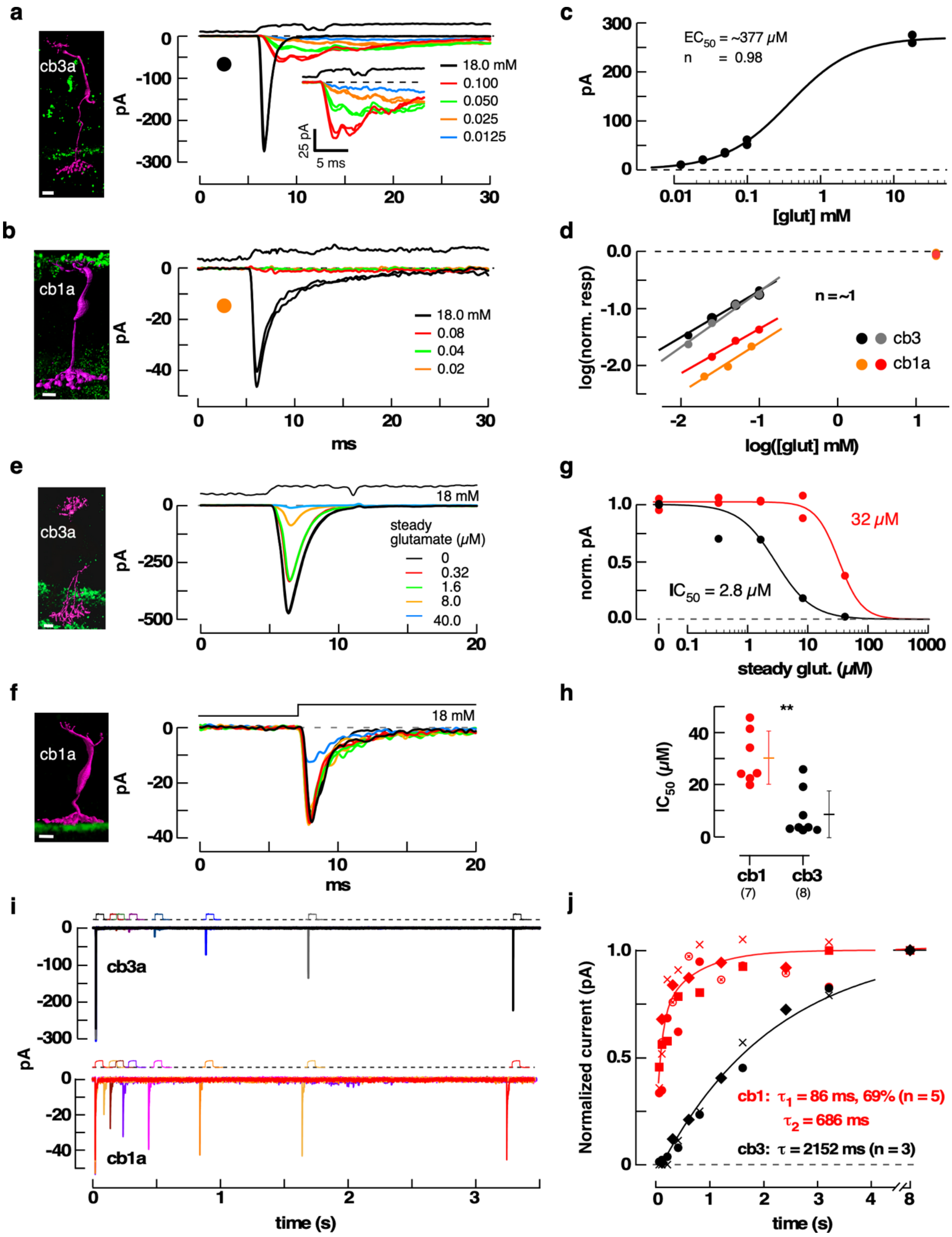

Supplementary Fig. 8. a.-d. Responses of cb1a and cb3 cell somas during rapid perfusion with low concentrations of glutamate (12.5 – 100 μM) with 18 mM glutamate as a reference. **a.** Confocal image (left) of a cb3a cell remnant (magenta) with an axon ramification below the upper ChAT band (green). Responses to concentrations of rapidly applied glutamate (right). *Inset.* Initial responses shown on expanded amplitude and time bases. Black trace, above, shows the measured solution junction potential resulting from the exchange of the rapid perfusate. Slight

"bounces" are due to perfusion pipette oscillation a few milliseconds after the end of the command step. **b.** Responses of a cb1a cell soma (cell remnant, magenta, Ribeye label, green) to glutamate concentration steps ranging from 20 to 80 μM with an 18 mM step as a reference. **c.** Peak response versus concentration plot for the data in *a*. Fit is to a Hill equation yielding an EC_{50} of ~377 μM and a Hill coefficient of 0.98. For the fit, the baseline was fixed at 0 and the maximum was fixed at 260 pA. Although missing data points in the mid-range, the fit parameters were consistent with those plotted in Fig. 5d. **d.** Log-log plot of peak response versus glutamate concentration for 4 somas. Colored circles in *a,b* indicate correspondence. The slope of the linear fit indicates a Hill coefficient of ~1. The relative glutamate sensitivities of cb3 and cb1a receptors are maintained at low concentrations of glutamate equivalent to those occurring at basal contacts during quantal release. **e.-h.** Steady-state inhibition (IC_{50}) was measured during a rapid step into a solution that contained 18 mM glutamate. Before the step, **e**. cb3a and **f**. a cb1a cell somas were bathed in a continuous steam of glutamate at concentrations between 0 and 40 μM. The time course of the solution change in *e* was measured with an open pipette tip. The time course was estimated in *f*. Cell remnants were counterstained with an antibody to ChAT (left). **g.** Plots of peak response versus steady glutamate concentration for the cells in *e* and *f* were fitted with a Hill curve to obtain the IC_{50}. An anomalously low peak response in 0.32 μM glutamate was omitted from the cb3a cell fit. **h.** Aggregate IC_{50} measurements (cb1a: IC_{50} = 30.4 ± 10.2 μM, Hill Coef. = 1.9 ± 1.0, n = 7 somas, mean ± S.D.; cb3: IC_{50} = 8.7 ± 9 μM, Hill Coef. = 2.8 ± 2.2, n = 8 somas, 5 cb3a and 3 cb3b; means are different p = 0.0004, two tailed unpaired t test). **i.** Paired pulse responses to 18 ms applications of 18 mM glutamate. **j**. Normalized amplitude of the second response in the pair was plotted against interpulse interval (ms) for 5 independently recorded cb1 and 3 independently recorded cb3 cell somas. Recovery was fitted with a single (cb3) or double (cb1) exponential curve. All experiments were performed in the presence of 35 μM GYKI53655. Scale = 5 μm.

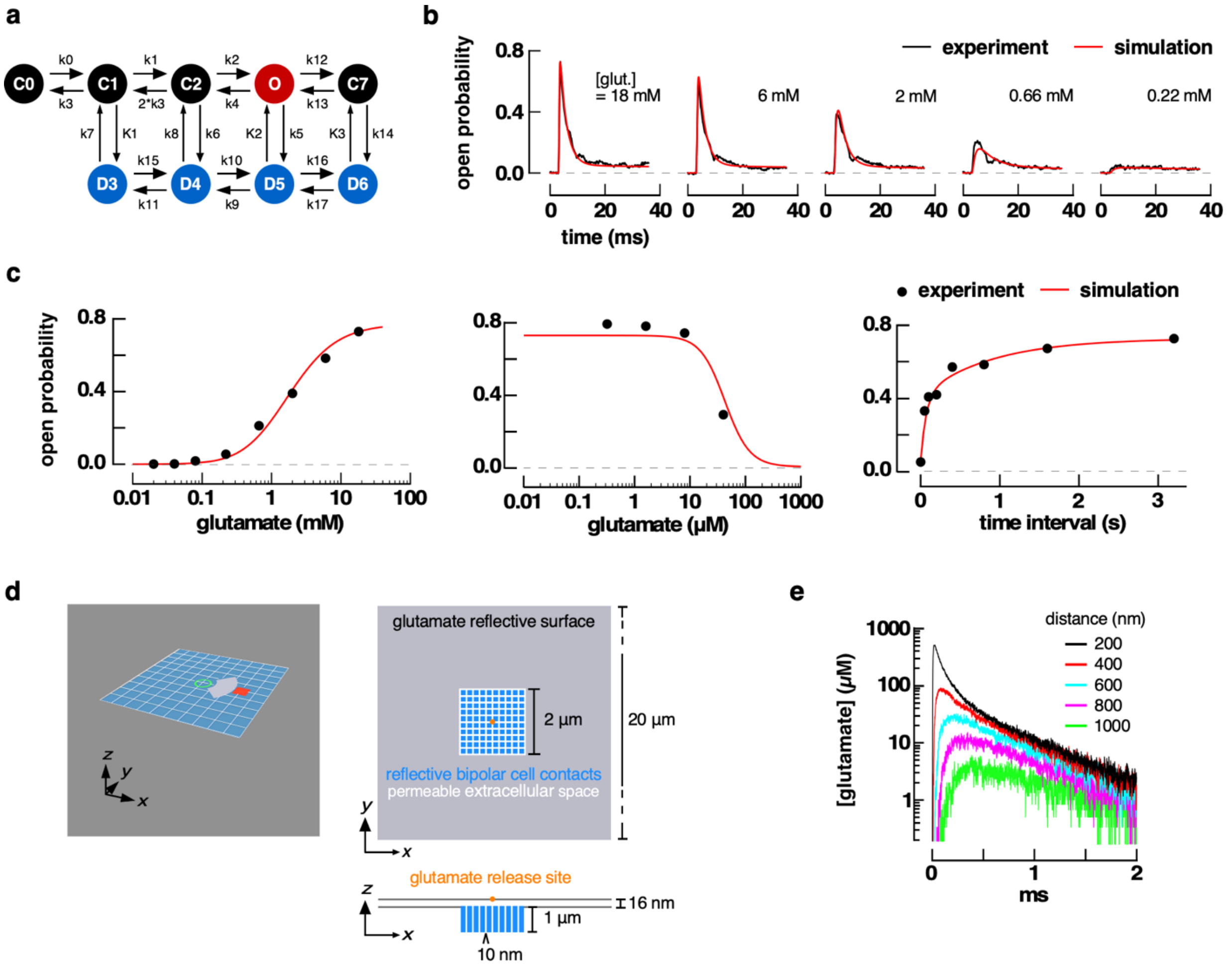

Supplementary Fig. 9. Cb1a cell receptor and synapse modeling. **a.** Rate constants in a 9-state receptor model[5] were obtained by Particle Swarm Optimization using the results from main text Fig. 5 and Supplementary Fig. 8 (see Supplementary Table 3). **b,c.** Model and experimental responses were compared during steps into different concentrations of glutamate. Model and experimental responses were also compared with respect to plots of EC_{50} and IC_{50}, and during recovery from desensitization. Results from the model had an EC_{50} = 1.83 mM (Hill coeff. n = 1.33), an IC_{50} = 43 μM (n = 1.92), and recovery τ_{fast} = 68.9 ms (49%) and τ_{slow} = 884.6 ms. **d.** Diagram of the synapse model constructed in Cellblender from different perspectives. The presynaptic surface is impermeable and reflective. The postsynaptic surface has a zone containing blue squares (impermeable and reflective) that are separated by white margins. The blue shape, a rectangular solid in 3 dimensions, represents the dendrites of basally contacting BCs whereas the white margins represent 16 nm extracellular spaces between dendrites where glutamate may leak out of the synaptic cleft. Otherwise, the area beyond the postsynaptic surface (gray) is impermeable. Receptor and transporter locations are color coded as in main text Fig. 7b. **e.** Plot of glutamate concentration as a function of time at various distances from a release site obtained from a Monte Carlo simulation. Release of 3000 glutamate molecules was instantaneous. Receptors and transporters were absent for this simulation.

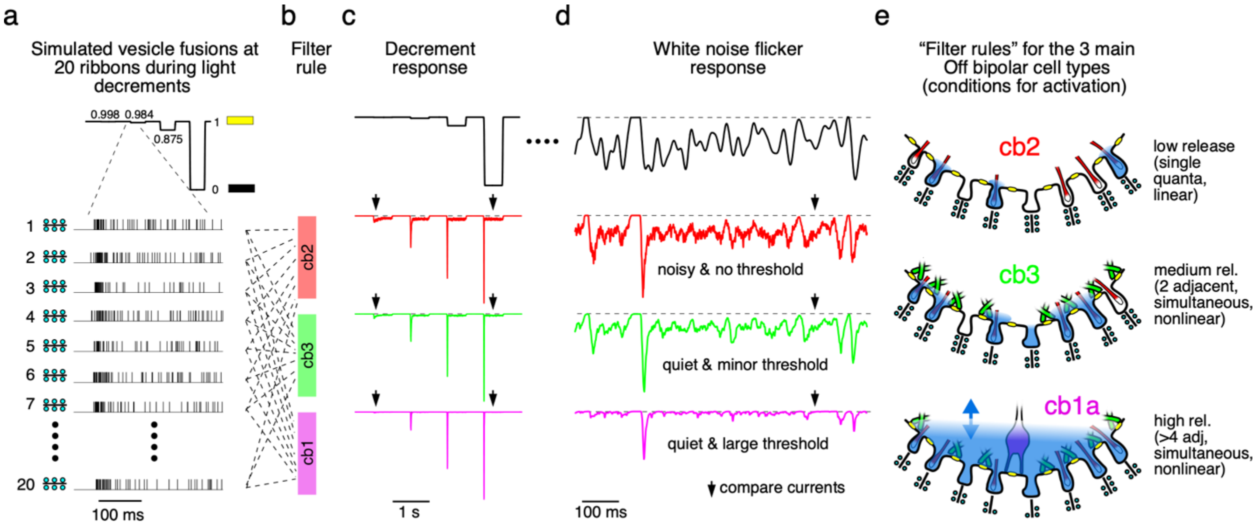

Supplementary Fig. 10. Simulated light responses and summary of results. **a.** In the model, a cone terminal contains 20 ribbons each with 28 independent vesicle docking sites. Fusion events at each ribbon active zone are symbolized by thin vertical lines. Individual empty docking sites are probabilistically refilled and primed at an average rate of 4 s^{-1}. The release probability of a primed vesicle is a function of light intensity. Light decrements that progressively increase by 8-fold are superimposed on a bright background (normalized to 1) that suppresses release. The light intensity trace was first passed through a Gaussian filter to simulate phototransduction (σ = 8.8 ms)[6]. Fusion events at the start of a decrement from 1 to 0.984 are shown on a faster time base. **b.** Ribbon fusion trains, binned into 1 ms intervals for summation, are passed through one of three filters. In the cb2 filter rule, release from all ribbons is summed with the exception that the number of transmitter quanta that can be summed in a time bin at an individual ribbon is capped at 5[7]. The cb3 filter rule is based on the model for basal contacts in Fig. 3c and assumes a full complement of 24 contacts per cone. A contact samples release from 3 surrounding invaginations and responds in 30% of the cases when one vesicle fuses in any of the three, with the remainder of the responses requiring that two or more vesicles fuse within the same time bin. Responses at each dendrite are then linearly summed at the soma. In the third filter rule, the cb1 cell samples from all release sites but requires 4 or more events at a dendrite to produce a response, followed by linear summation of transduced events at the soma. **c.** Decrement step responses. The filtered output from *a*, a modified train of transduced events, was convolved with the quantal response profile for each BC type and the results are shown. Note the minimal steady response in the cb1 cell (arrows). **d.** 100 Hz bandwidth-limited white noise was filtered by phototransduction, converted to a sequence of vesicle fusions at each ribbon, and then processed by each filter rule. The output of the filter was convolved with the quantal response profile for each BC type. Traces are each an average of 8 independent runs. The cb2 response is noisy and has no threshold, the cb3 response has reduced noise due to averaging and a minor threshold, while the cb1 simulation retains large transient responses while thresholding-out smaller events. **e.** Graphical illustration of the different operating ranges of cb2, cb3, and cb1a cells. Cb2: The glutamate released when a single vesicle fuses (low release conditions) in an invagination reliably activates GluA4 receptors on the invaginating dendrite. Glutamate spills out of the invagination and onto the local basal surface where it is buffered by transporter glutamate binding sites. Dendrites respond only to release from the local ribbon. Cb3: Release of glutamate from two or more vesicles at neighboring invaginations (medium release conditions) begins to saturate local transporter binding sites and enables responses in relatively high affinity GluK1 subunit-containing receptors located on dendrites at the base of the terminal. Cb1a: A combination of diffusion distance, high receptor EC_{50}, and transporter glutamate buffering creates a distinct response threshold. The centrally located dendrite responds to the "tidal" flow of glutamate within the terminal cap region resulting from multiple, simultaneous release events (high release conditions).

Supplementary Tables

Supplementary Table 1. Spatial profile of GluK1 distribution beneath cone terminal.

The column items are the features of the elliptical profile obtained from the five STED images shown in Supplementary Fig. 6b. "Circularity" is the ratio of the minor axis to the major axis of the detected elliptical profile; the closer the ratio is to 1, the closer it is to a perfect circle. The intensity in a pixel is given by the fraction of the summed images that contain a process at that position.

Data label	Circularity	Pixel average (%) in	
		annulus	dimple
#0	0.767	60.92	49.79
#1	0.871	67.50	53.74
#2	0.821	85.58	83.40
#3	0.982	73.69	67.13
#4	0.963	67.74	61.00
Mean	0.881	71.09	63.01
SD	0.092	9.28	13.21

Supplementary Table 2. Centroid displacement.

The data labeled "green" and "red" correspond to the filled-in green and red circles in Supplementary Fig. 6d. The data labeled "average" corresponds to the open circles in Supplementary Fig. 6d.

Data label	distance (nm)	direction (deg.)
green #0	17.53	120.34
green #1	15.55	277.81
green #2	8.16	84.05
green #3	42.72	210.41
green #4	29.57	255.72
average	13.12	220.30
red #0	47.42	333.04
red #1	70.81	87.65
average	33.41	47.48

Supplementary Table 3. Rate constants in cb1a and cb2 models.

The unit of the rate constants is $M^{-1}s^{-1}$ or s^{-1}. In order to establish the microscopic reversibility of the model, let $K_1 = (k_7*k_1*k_6*k_{11})/(k_{15}*k_8*k_3)$, $K_2 = (k_5*k_9*k_8*k_2)/(k_4*k_6*k_{10})$ and $K_3 = (k_{14}*k_{17}*K_2*k_{12})/(k_{13}*k_5*k_{16})$.

Parameters	Value for cb1a	Value for cb2
k_0	4.182E+06	1.057E+08
k_1	2.091E+06	7.765E+03
k_2	8.426E+03	1.723E+04
k_3	5.307E+03	2.093E+03
k_4	4.821E+02	3.734E+03
k_5	9.547E-01	1.865E+01
k_6	1.533E+02	5.132E+03
k_7	2.317E+03	5.192E+00
k_8	3.403E+00	4.665E+01
k_9	6.378E+01	4.209E+02
k_{10}	7.494E+01	4.764E+02
k_{11}	3.308E+02	3.486E+03
k_{12}	3.994E+02	6.846E+02
k_{13}	2.354E+01	9.047E+01
k_{14}	4.780E+00	1.878E+01
k_{15}	1.807E+07	7.777E+06
k_{16}	5.316E+04	1.034E+04
k_{17}	8.739E+02	6.363E+02

References

1 Grabner, C. P. & Moser, T. Individual synaptic vesicles mediate stimulated exocytosis from cochlear inner hair cells. *Proceedings of the National Academy of Sciences of the United States of America* **115**, 12811-12816 (2018).

2 Gameiro, A., Braams, S., Rauen, T. & Grewer, C. The discovery of slowness: low-capacity transport and slow anion channel gating by the glutamate transporter EAAT5. *Biophysical Journal* **100**, 2623-2632 (2011).

3 Rowan, M. J., Ripps, H. & Shen, W. Fast glutamate uptake via EAAT2 shapes the cone-mediated light offset response in bipolar cells. *The Journal of physiology* **588**, 3943-3956 (2010).

4 Wassle, H., Puller, C., Muller, F. & Haverkamp, S. Cone contacts, mosaics, and territories of bipolar cells in the mouse retina. *The Journal of Neuroscience* **29**, 106-117 (2009).

5 Hausser, M. & Roth, A. Dendritic and somatic glutamate receptor channels in rat cerebellar Purkinje cells. *The Journal of Physiology* **501**, 77-95 (1997).

6 Kraft, T. W. Photocurrents of cone photoreceptors of the golden-mantled ground squirrel. *The Journal of Physiology* **404**, 199-213 (1988).

7 Grabner, C. P., Ratliff, C. P., Light, A. C. & DeVries, S. H. Mechanism of High-Frequency Signaling at a Depressing Ribbon Synapse. *Neuron* **91**, 133-145 (2016).
