# Supplementary material for: Mechanisms of simultaneous linear and nonlinear computations at the mammalian cone photoreceptor synapse
Source: Nat Commun. 2023 Jun 16;14:3486. doi: 10.1038/s41467-023-38943-2 (PMC10276006; doi:10.1038/s41467-023-38943-2)
Supplement: Supplementary file 2 — Reporting Summary [file 41467_2023_38943_MOESM2_ESM.pdf]

nature portfolio

Corresponding author(s): Steven H. DeVries

Last updated by author(s): March 26, 2023

Reporting Summary

Nature Portfolio wishes to improve the reproducibility of the work that we publish. This form provides structure for consistency and transparency in reporting. For further information on Nature Portfolio policies, see our Editorial Policies and the Editorial Policy Checklist.

Statistics

For all statistical analyses, confirm that the following items are present in the figure legend, table legend, main text, or Methods section.

n/a	Confirmed	
☐	☒	The exact sample size (*n*) for each experimental group/condition, given as a discrete number and unit of measurement
☐	☒	A statement on whether measurements were taken from distinct samples or whether the same sample was measured repeatedly
☐	☒	The statistical test(s) used AND whether they are one- or two-sided<br>*Only common tests should be described solely by name; describe more complex techniques in the Methods section.*
☒	☐	A description of all covariates tested
☒	☐	A description of any assumptions or corrections, such as tests of normality and adjustment for multiple comparisons
☐	☒	A full description of the statistical parameters including central tendency (e.g. means) or other basic estimates (e.g. regression coefficient) AND variation (e.g. standard deviation) or associated estimates of uncertainty (e.g. confidence intervals)
☐	☒	For null hypothesis testing, the test statistic (e.g. *F*, *t*, *r*) with confidence intervals, effect sizes, degrees of freedom and *P* value noted<br>*Give P values as exact values whenever suitable.*
☒	☐	For Bayesian analysis, information on the choice of priors and Markov chain Monte Carlo settings
☒	☐	For hierarchical and complex designs, identification of the appropriate level for tests and full reporting of outcomes
☒	☐	Estimates of effect sizes (e.g. Cohen's *d*, Pearson's *r*), indicating how they were calculated

Our web collection on statistics for biologists contains articles on many of the points above.

Software and code

Policy information about availability of computer code

Data collection	Patchmaster 2x73.3 (HEKA, Lambrecht, Pfalz, Germany); IgorPro 6.37 with Universal ITC18 XOP vers. 7.6 (Wavemetrics, Oswego, OR). Custom acquisition code is not general purpose but rather specifically configured for the in-lab physiology recording equipment and individual experimental routines. STED images were acquired on a Leica SP8 3D-STED microscope using LASX software (3.5.7.23225).
Data analysis	Patchmaster 2x73.3 (HEKA, Lambrecht, Pfalz, Germany) and Origin v. 9.3 (OriginLab; Northampton. MA, USA) were used for analysis of capacitance measurements. Image J 1.51 was used for the preliminary analysis of EM and STED images. Images were denoised using FIJI CSBDeep Noise2Void (0.8.6.), deconvolved using Imaris 9.9.0 with ClearView Deconvolution 9.8 , segmented with Blender 2.93, and further analyzed with Python 3.7.4 executing in the JupyterLab 1.1.4 environment with the addition of NumPy 1.17.2 and scikit-image 0.15.0 libraries. CanvasXDraw 7.0.3 was used for the centroid analysis. Computer code (IgorPro 8.0, Wavemetrics, Oswego, OR) for the subroutines used to characterize epscs in cb1, cb2, and cb3 Off bipolar cells and for modeling synapse probabilities is available on Zenodo via the Dryad link doi:10.5061/dryad.0p2ngf25g. Rate constant optimization was performed using PySwarms 1.3.0 and synapse modeling was performed using MCELL 3.4 and CellBlender 1.0.1. Statistical analysis was performed with GraphPad.com/Resources .

For manuscripts utilizing custom algorithms or software that are central to the research but not yet described in published literature, software must be made available to editors and reviewers. We strongly encourage code deposition in a community repository (e.g. GitHub). See the Nature Portfolio guidelines for submitting code & software for further information.

Data

Policy information about availability of data

All manuscripts must include a data availability statement. This statement should provide the following information, where applicable:

- Accession codes, unique identifiers, or web links for publicly available datasets
- A description of any restrictions on data availability
- For clinical datasets or third party data, please ensure that the statement adheres to our policy

Source data is provided in a spreadsheet for all electrophysiological traces and graphs in figures. Raw image stacks, deconvolved image stacks, and surface fits are available at Dryad link doi:10.5061/dryad.0p2ngf25g. Additional raw data will be shared upon request.

Human research participants

Policy information about studies involving human research participants and Sex and Gender in Research.

Reporting on sex and gender	n/a
Population characteristics	n/a
Recruitment	n/a
Ethics oversight	n/a

Note that full information on the approval of the study protocol must also be provided in the manuscript.

Field-specific reporting

Please select the one below that is the best fit for your research. If you are not sure, read the appropriate sections before making your selection.

☒ Life sciences ☐ Behavioural & social sciences ☐ Ecological, evolutionary & environmental sciences

For a reference copy of the document with all sections, see nature.com/documents/nr-reporting-summary-flat.pdf

Life sciences study design

All studies must disclose on these points even when the disclosure is negative.

Sample size	We did not perform a power analysis to determine sample size. Rather, sample sizes reflect those that are both standard for the field and used in our previous studies (e.g., Grabner et al, 2016).
Data exclusions	A preliminary analysis was performed on all electrophysiological data. Criterion for the exclusion of individual experiments are described and quantified in Supplementary information Figure 2, the legend of figure 2, and in Methods. Criteria are both standard to the field (e.g., excessive "leak"current) and based on deviation from the standards developed from the global data set involving hundreds of independent cone to Off bipolar cell pair recordings over several decades (e.g., for mean and maximal peak epsc responses and rise rates). Exclusions were applied consistently across the sample. Additionally, an experiment was excluded if data was not reproducible within the experiment (e.g., lack of recovery of cone tpsc responses after TBOA exposure; loss of the soma before 2 full concentration-response runs were completed during rapid perfusion).
Replication	Experiments were replicated as noted in the figures/text of the study. Exemplar results presented in the main text align with the mean behavior of the sample unless otherwise indicated. The thirteen-lined ground squirrel hibernates in the wild and will occasionally enter torpor when maintained at room temperature in the animal facility on a consistent 12:12 hr light cycle. Only active animals were selected for experimentation irrespective of season. Authentic hibernation can have a small effect on photoreceptor synapse function (Mehta et al, 2013) including a reduction in peak epsc amplitude and the rate of steady vesicle turnover at the ribbon. As these experiments targeted small epscs, no seasonal effects were noted. The data set spans more then 10 years during which recordings were performed throughout the year without noticeable differences in the response properties measured.
Randomization	Animals are wild caught in a roughly equal male to female ratio. New animals were introduced into the colony yearly and maintained for up to 3 years. Except for excluding potentially torpid animals during the winter months, adult animals were randomly selected one at a time for experimentation without regard for age or sex. While some experiments had internal controls (e.g., those involving TBOA), for others, there was no formal control group. Rather the responses at cone to cb1, cb2, or cb3 cell synapses were contrasted with each other. The identity of the recorded bipolar cell could not be known in advance of an experiment and at most three pairs were recorded from the same animal. Thus each 'n' contains results from many different randomly-selected animals.
Blinding	Pre- and postsynaptic event amplitudes were assigned based on an automated computer algorithm that was invariant with respect to bipolar cell type. Bipolar cell type was only confirmed after data analysis by reviewing images collected either on the physiology rig at the end of an experiment or by confocal imaging of fixed and labeled tissue, or both. Type was assigned based on published criteria from the lab (Light et

al, 2012). Nonetheless, because there was only one person in the lab during this period able to both perform experiments and analyze the data, the investigator could not be fully blinded to a preliminary identification of the cell type prior to analysis.

Reporting for specific materials, systems and methods

We require information from authors about some types of materials, experimental systems and methods used in many studies. Here, indicate whether each material, system or method listed is relevant to your study. If you are not sure if a list item applies to your research, read the appropriate section before selecting a response.

Materials & experimental systems

n/a	Involved in the study
☐	☒ Antibodies
☒	☐ Eukaryotic cell lines
☒	☐ Palaeontology and archaeology
☐	☒ Animals and other organisms
☒	☐ Clinical data
☒	☐ Dual use research of concern

Methods

n/a	Involved in the study
☒	☐ ChIP-seq
☒	☐ Flow cytometry
☒	☐ MRI-based neuroimaging

Antibodies

Antibodies used

Anti-PSD95 PDZ domain, SySy 124014, guinea pig
Anti-SLC1A7 (EAAT5), Sigma, HPA049124, rabbit
Anti-CtBP2 (C-16), Santa Cruz Biotechnology, sc-5967, goat
Anti-GluA4, EMD Millipore, AB1508, rabbit
Anti-GluK1 (E-12), Santa Cruz Biotechnology, sc-393420, mouse monoclonal
Anti-ChAT, Chemicon, #AB144P, goat
Anti-GFP, Abcam, ab13970, chicken
Anti-Bassoon, SySy 141016, chicken
Anti-Alexa Fluor 405/Cascade Blue, Thermofisher, A-5760, rabbit
Donkey anti-mouse, JacksonImmuno, #715-005-151
Donkey anti-rabbit, JacksonImmuno, #711-005-152
Donkey anti-goat, JacksonImmuno, #703-005-155
Donkey anti-guinea pig, JacksonImmuno, #706-005-148
Donkey anti-chicken, JacksonImmuno, #705-005-147

Validation

These commercially available antibodies are widely used in the field and have been validated in retinas from multiple species.

Animals and other research organisms

Policy information about studies involving animals; ARRIVE guidelines recommended for reporting animal research, and Sex and Gender in Research

Laboratory animals

Most of the laboratory animals in this study were wild-caught by TLS (Bartlett, IL) and maintained in a colony in the Center for Comparative Medicine at Northwestern University Feinberg School of Medicine for 1-3 years at 72F on 12:12 hr light:dark cycle. A small number of animals were bred in captivity. Experiments were performed on adult animals of either sex.

Wild animals

Wild animals were caught in the field by a state-licensed trapper. Following introduction into the animal colony, animals were treated for ectoparasites and quarantined for a minimum of 3 months.

Reporting on sex

Experiments used adult ground squirrels of both sexes equally. No consistent differences were noted in the results related to the sex of the animal.

Field-collected samples

No field collected samples were used in this study

Ethics oversight

All animal procedures were approved by the Northwestern University Institutional Care and Use Committee (IACUC).

Note that full information on the approval of the study protocol must also be provided in the manuscript.
