# Supplementary material for: Mechanisms of simultaneous linear and nonlinear computations at the mammalian cone photoreceptor synapse
Source: Nat Commun. 2023 Jun 16;14:3486. doi: 10.1038/s41467-023-38943-2 (PMC10276006; doi:10.1038/s41467-023-38943-2)
Supplement: Supplementary file 3 — Description of Additional Supplementary Files [file 41467_2023_38943_MOESM3_ESM.pdf]

Description of Additional Supplementary Files

Supplementary Movie 1
Description: 3D STED image corresponding to Fig. 7a showing labels for PSD95 (red), EAAT5 (cyan), GluA4 (yellow), and GluK1 (magenta).
